# The Active Side of Stereopsis: Fixation Strategy and Adaptation to Natural Environments

**DOI:** 10.1038/srep44800

**Published:** 2017-03-20

**Authors:** Agostino Gibaldi, Andrea Canessa, Silvio P. Sabatini

**Affiliations:** 1 Physical Structure of Perception and Computation Group, Department of Informatics, Bioengineering, Robotics and System Engineering, University of Genoa, 16145, Genoa, Italy

## Abstract

Depth perception in near viewing strongly relies on the interpretation of binocular retinal disparity to obtain stereopsis. Statistical regularities of retinal disparities have been claimed to greatly impact on the neural mechanisms that underlie binocular vision, both to facilitate perceptual decisions and to reduce computational load. In this paper, we designed a novel and unconventional approach in order to assess the role of fixation strategy in conditioning the statistics of retinal disparity. We integrated accurate realistic three-dimensional models of natural scenes with binocular eye movement recording, to obtain accurate ground-truth statistics of retinal disparity experienced by a subject in near viewing. Our results evidence how the organization of human binocular visual system is finely adapted to the disparity statistics characterizing actual fixations, thus revealing a novel role of the active fixation strategy over the binocular visual functionality. This suggests an ecological explanation for the intrinsic preference of stereopsis for a close central object surrounded by a far background, as an early binocular aspect of the figure-ground segregation process.

The human visual system is characterized by two frontal eyes that actively explore the visual space with highly overlapped visual fields. Due to the horizontal separation of the two eyes, an object in space projects onto slightly different locations in the left and right retinas, and this shift is defined as the retinal binocular disparity. Retinal disparity is interpreted by the visual system to obtain stereopsis, which is the prominent visual feature for depth perception in near viewing. To this aim, the visual system has to determine which points in the two retinal images correspond to the same object in the scene, *i.e.* to solve the stereo correspondence problem. The primary visual cortex (V1) is the first location along the visual cortical pathway where the information from the two eyes is integrated, and is thus considered the neural front-end of disparity encoding and interpretation. The cortical cells in V1 are sensitive to different magnitudes and orientations of binocular disparity^1^,^2^. This distributed representation of binocular information is subsequently used by higher visual areas for understanding the three-dimensionality of the scene^3^,^4^.

The processing of retinal disparity is a computationally demanding task, since a single natural scene has unpredictable complex and cluttered three-dimensional (3D) structure. Nonetheless, natural environments present statistical regularities^5^,^6^. The efficient coding theory predicts that the visual system shall exploit these regularities for an optimal allocation of the required computational resources and to facilitate perceptual judgments^7^,^8^,^9^. Psychophysical experiments thus showed how the binocular visual system likely adapts to the 3D structure of natural environments, making the region of single vision and precise stereopsis coincident with particular surface shape and orientation that are behaviorally advantageous, as the ground floor^10^,^11^,^12^,^13^ or the close working environment^10^,^14^. Coherently, also the functional characteristics of the underlying neural substrate have been shown to adapt to the likely distributions of retinal disparities occurring in natural scenes. In the first place, the specialization for horizontal disparity in V1 was related to the geometry of the binocular visual system^15^. The statistics of disparity in natural environments have been shown to correlate with the tuning of complex cells in V1^16^,^17^. More specifically, the anisotropy of disparity tuning in V1 area closely resembles the retinotopic disparity statistics of natural environments^14^, evidencing a direct relation between the visual system and the environment where it develops.

As a complicating factor, our eyes constantly shift position to explore the visual scene, requiring to recompute retinal disparity at each fixation. In order to collect statistics that closely represent the actual experienced disparities, eye movements were simulated on synthetic virtual scenes^18^,^19^, or natural scenes^16^,^20^. To this aim, considering the active fixation strategy of the subject is a mandatory step to obtain realistic disparity statistics, since fixations are neither randomly nor evenly distributed within the environment. Specifically, actual human fixation is preferentially directed towards close targets within the visual scene^21^,^22^,^23^, and behaviorally significant points more likely attract our gaze. This strategy allows us gaining accurate visual information about the environment and eventually to plan and accomplish different tasks, such as object recognition, grasping, and manipulation^24^. Even if the 3D environment is characterized by intrinsic statistical properties, it is the active fixation strategy that mediates its visual experience. Specifically, from an active observer perspective, it occurs that the fixated object is more frequently a close central object surrounded by a far background. This effect additionally conditions the geometrical relation between the left right retinas^14^, thus bounding the search zone for disparity in a way than depends on both the task at hand and the adopted strategy to accomplish it.

In the present work, we specifically investigate the possible role of active fixation strategy on the binocular visual system. Our methodology exploits a unique virtual reality setup that combines naturalistic stereoscopic stimulation with binocular eye movement recording. Since stereopsis has been proven relevant to depth perception mainly in close viewing^25^, we used 3D models of natural working environments bounded within peripersonal space (distances <2 m). Whereas gathering disparity statistics for human observers interacting with real environments^14^ is an ideal condition to allow for freedom of movements and naturalness of the performed tasks, virtual reality setups present valuable advantages^26^. The high degree of control characterizing our methodology, allowed us to obtain *ground-truth* disparity information characterized by high accuracy and precision that would not be possible with other methods. Moreover, the disparity patterns corresponding to actual subjects’ fixations were computed by mimicking the natural human eye posture^27^,^28^,^29^.

The obtained disparity distributions were analyzed with respect to random fixations of a virtual observer, evidencing a direct influence of the active fixation strategy over the sensory experience of the 3D environment. Furthermore, the actual binocular exploration of a human subject was shown to provide a better accounting for neurophysiological and psychophysical data than random fixations. The mechanism of stereopsis thus exhibit a fine retinotopic adaptation to the working environment, evidencing how fixation strategy consequently conditions the functional characteristics of the visual system. This suggests an ecological explanation of the intrinsic preference of the early neural mechanism of stereopsis for a close central object surrounded by a far background, specialized in higher visual areas, as a binocular aspect of the figure-ground segregation process.

## Results

### Statistics of the 3D Visual Space: Distribution of Retinal Disparities

**Figure 1:**
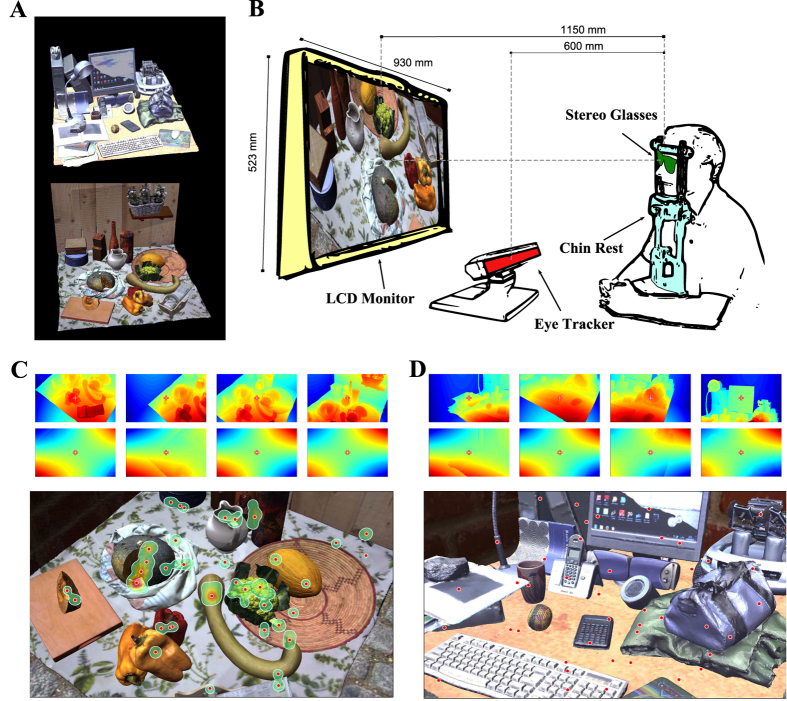
(**A**) Three-dimensional environments. Sketch representing the two naturalistic virtual scenes used: an office desk (top) and a kitchen table (bottom). (**B**) Stereoscopic experimental setup. Representation of the setup for computing binocular fixations on subjects. (**C**) Fixation density map. Heatmap (contour lines) and fixation points (red dots) superimposed to the cyclopean image for a subject exploring a binocular image (bottom). and horizontal and vertical disparity patterns for selected fixation points (top). (**D**) Random fixations. Random fixation points (red dots) (bottom), and selected horizontal and vertical disparity patterns (top).

**Figure 2:**
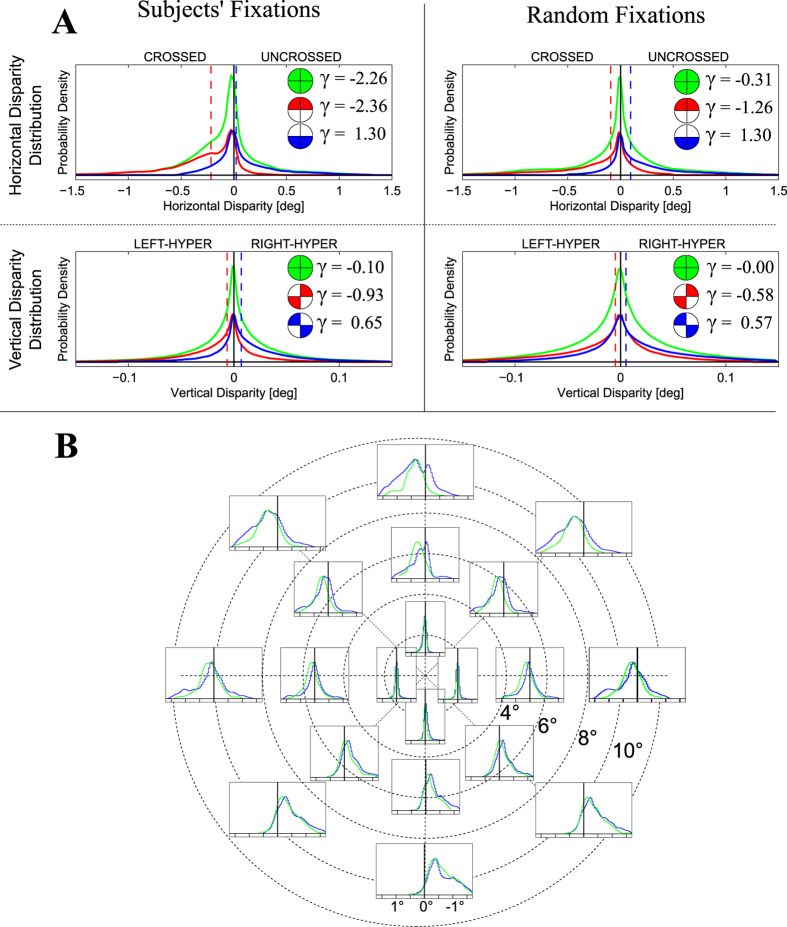
(**A**) Disparity distribution on different quadrants. Horizontal (top) and vertical (bottom) disparity distribution for subjects’ (left) and random fixations (right), computed over the whole field of view (green). For horizontal disparity, the distribution has been recomputed separating the upper (red) by the lower (blue) hemifields. For vertical disparity the distribution has been recomputed separating the top-right and bottom-left quadrants (red) by the upper-left and lower-right (blue). According to our notation, crossed horizontal disparities and right-hyper vertical disparity are positive, while uncrossed horizontal disparities and left-hyper vertical disparities are negative. The vertical dashed lines represent the median of the considered distributions. The insets represent the separation, together with the skewness *γ* of the corresponding distribution. (**B**) Topographic representation of the disparity distribution. Horizontal disparity distribution computed at different image locations (*i.e.* pixel) for random fixations (blue) and subjects’ actual fixations (green): three different eccentricities (1°, 5°, 10°) and eight different orientations.

**Figure 3:**
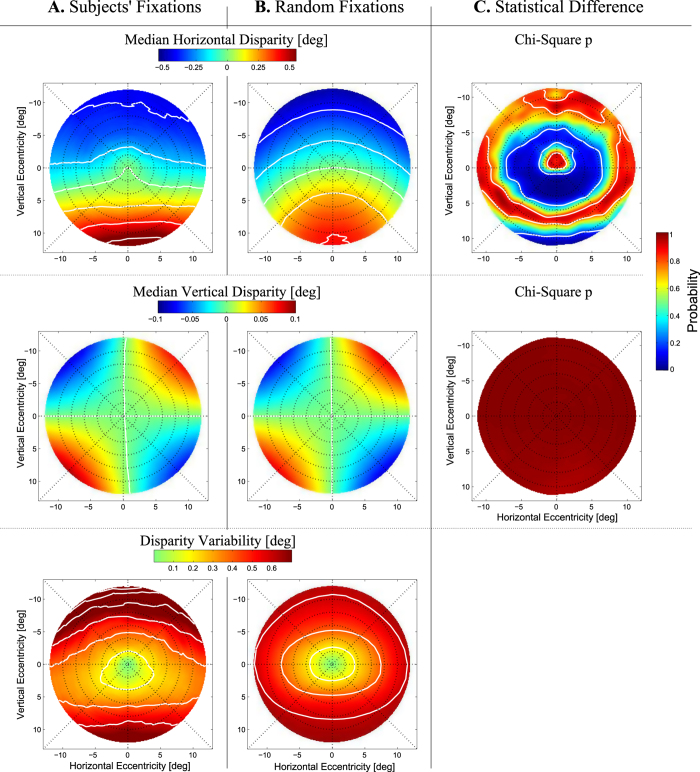
(**A**) Disparity patterns for subjects’ fixations. Retinotopic patterns of disparity distributions derived from subjects’ fixations: median horizontal (top) and vertical (middle), and standard deviation (bottom). The white lines represent iso-contour levels at steps of 0.1°. (**B**) Disparity patterns for random fixations. Same representation as in panel A, but derived from random fixations. According to our notation, crossed horizontal disparities and right-hyper vertical disparity are positive (red), while uncrossed horizontal disparities and left-hyper vertical disparities are negative (blue). (**C**) Distance between the two distributions. *χ*^2^ probability (*p*-value) of the Mahalanobis distance between the random and the subjects’ fixations distributions, for horizontal (left) and vertical (right) disparities.

We first collected the binocular eye gaze data of human subjects engaged in exploratory fixation tasks in peripersonal space. These data where then used to generate the corresponding ground-truth horizontal and vertical disparity patterns experienced at fixations (see Methods and ref. 30 and 31, for further details). Next, we generated random fixations of a virtual observer exploiting a stochastic sampling of gaze distribution measured on subjects (see Methods, and Fig. 1). We thus obtained two distinct disparity distributions, one for subjects’ fixations and one from random fixations. The resulting probability distributions were plotted 1) over the central field of view (first 10° from the fovea), 2) grouping different quadrants of the visual field (Fig. 2A), and 3) for different selected retinal locations (Fig. 2B). To provide a retinotopical characterization of disparity distributions, we derived the central value of the distribution (first moment), and its variability (second moment). The results were plotted for the subjects’ and the random fixations as retinotopic maps in Fig. 3A,B (see also Supplementary Fig. S1), together with a *p*-value map for statistical comparison in Fig. 3C. Crossed horizontal disparities (objects closer than fixation point) are defined positive and are displayed in red, while uncrossed disparities are in blue. Similarly, for vertical disparities (expressed as elevation-longitude^32^) red colors represent right-hyper while blue colors stand for left-hyper disparities.

The obtained results generally reflect the distribution of the tuning characteristics of macaque V1 area^33^,^34^,^35^,^36^, in accordance with previous studies that consider the central part of the field of view^15^,^16^,^18^,^37^. Though, it is worth noticing that the approaches that rely on the epipolar geometry^15^,^37^ or on synthetic worlds^18^ resulted in isotropic patterns over the retinal plane, which are not likely to properly reflect what occurs in natural scenes. Normal working/living environments are behaviorally close to top-back slanted surfaces^5^,^10^,^14^, which are uncrossed (*i.e.* further than the fixation point) in the upper visual field and crossed (*i.e.* closer than the fixation point) in the lower one. Our data show a pronounced difference between the upper and the lower hemifields for both the disparity distribution (Fig. 2A, top) and the median horizontal disparity patterns (Fig. 3A,B, top), in accordance with such structure of the natural environment. Considering the median vertical disparity, the distribution over the top-right and bottom-left quadrants presents the typical skewness towards positive values, while in the upper-left and lower-right quadrants it presents a skewness towards negative values (Fig. 2A, bottom, and Fig. 3A,B, middle), according to the commonly disparity experienced in vergent geometry^14^,^38^. A further consideration can be made about the second moment of the disparity distribution, *i.e.* its variability. By definition, the fixated object projects to the foveas of both eyes, resulting in small disparity range centered about zero, whereas disparity is less deterministic as we move peripherally^14^,^16^,^18^. Accordingly, our results provide a variability that is close to zero in a foveal area and increases with retinal eccentricity (Fig. 3A,B, bottom).

An essential difference stands out by comparing the disparity statistics resulting from subjects’ and random fixations, which, to the best of our knowledge, has never been evidenced before. With respect to random fixations, subjects’ fixations result in a horizontal median pattern characterized by a peculiar feature. The central area of visual field (≈2°) is at null or small disparities, surrounded by a region at negative disparities (Fig. 3A,B, top). Accordingly, the two distributions tend to be significantly similar about fixation and at large eccentricities (Fig. 3C), particularly in the upper hemifield (see Fig. 2). Subjects’ actual fixations yield a meaningful skewness to uncrossed horizontal disparities^14^,^16^, differently from random fixations^18^,^37^. Besides, vertical disparity is much less affected by the structure of the environment than the horizontal disparity. Accordingly, no significant differences are present between subjects’ and random fixations (Fig. 2A, top), both considering the distribution over the four quadrants of the visual field^14^, and the disparity patterns (Fig. 3).

Our results suggest a general tendency of active exploratory behavior, which is not simply to fixate the closest points of a 3D scene^21^,^22^,^23^. A fixating observer is behaviorally attracted by significant points such as the objects he is manipulating^24^, directing his gaze toward both the object center of mass^39^ and its upper region^40^,^41^. Accordingly, the central field of view experiences small disparities (see Fig. 3), corresponding to the *close* fixated object, while outside the object’s projection, the *far* background results in uncrossed disparities, particularly in the upper hemifield (see Fig. 2A).

Summarizing, subjects’ and random fixations provide qualitative similar disparity distributions, resembling the structure of the observed world. Yet, active exploratory fixations provide distributions that significantly *deviate* from those resulting by random fixations, particularly about the fovea. This evidence how the sensory experience of the 3D environment is actively mediated by the fixation strategy. In the following we will analyze the possible relations of disparity statistics with the functional and perceptual properties of the human visual system. We will focus, in the first place, on the first-order regularity of retinal disparity in order to predict plausible patterns of retinal correspondences, as well as the shape of the horopter, for a direct comparison with empirical measurements achieved on humans.

### Retinal Corresponding Points and the Empirical Horopter

The geometry of binocular vision allows for a theoretical characterization of the binocular correspondence problem, through two reference concepts, the geometric horopter and Panum’s fusional area. While we will return on the latter in the next section, the geometric horopter is the locus of points in three-dimensional (3D) space whose projections fall on the two retinas in geometrically corresponding points, *i.e.* with zero disparity^11^,^25^. The geometric horopter is composed of two parts (see Supplementary Fig. S2): 1) the horizontal horopter is a circle passing through the nodal point of the two eyes and the fixation point, the Vieth-Müller circle; 2) the vertical horopter is a line orthogonal to the fixation plane, lying in the median plane of the head, and passing through the Vieth-Müller circle.

Actually, psychophysical experiments showed that binocular vision functionally relies on empirical correspondences between the left and right retinal points, which deviates from the geometric ones. This phenomenon extends the geometric horopter to a surface, the empirical horopter, as the surface of optimal depth perception^42^. The perceptual advantage is to adapt depth perception to the 3D structure of natural environments. The deviations from the horizontal and vertical geometric horopter directly results from the Hering-Hillebrand and Helmholtz shear deviations of retinal corresponding points, respectively.

Along the horizontal retinal meridian, points in the temporal hemiretinas are relatively compressed with respect to the corresponding points in the nasal hemiretinas, yielding the Hering-Hillebrand deviation. This results in a bias towards uncrossed disparities to the left and right of fixation, and it is usually represented by a factor *H*, which defines the disparity relative to the geometric horopter (see Methods). The horizontal horopter tends to be less concave than the Vieth-Müller circle in the near viewing, flat at the abathic distance (≈1m), and more convex at far distances^10^,^12^,^25^,^43^,^44^,^45^,^46^. Considering the vertical retinal meridian, empirical corresponding points are anisotropic, presenting a shift towards uncrossed disparities in the upper hemiretina, and towards crossed disparities in the lower one. The Helmholtz shear deviation describes this anisotropy, and it is usually represented by the shear angle between the two corresponding vertical meridians. The resulting vertical horopter is tilted top away in the median plane and its slant increases with the fixation distance^10^,^11^,^12^,^13^,^14^,^47^,^48^.

#### Predicted Corresponding Points

**Figure 4:**
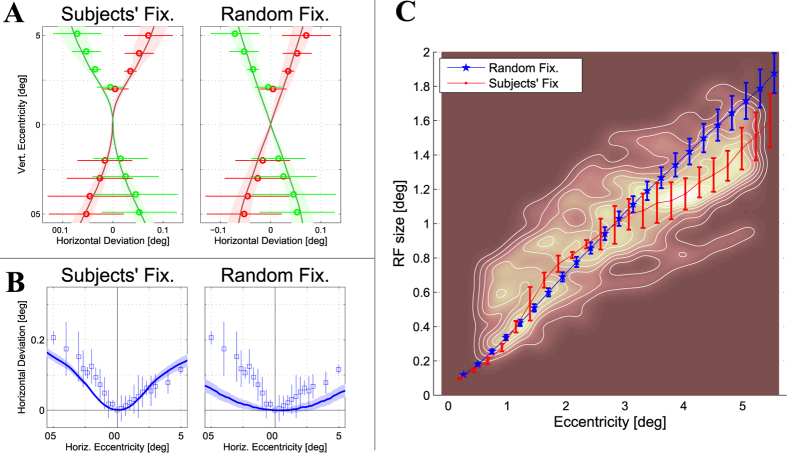
(**A**) Helmholtz shear deviation. Helmholtz shear deviation inferred by the obtained disparity distribution (solid lines) for the subjects’ (left) and random (right) fixations. The solid lines and the colored regions represent the mean and standard deviation computed among the four subjects (left), while for random fixations (right)they represent the mean and the 95% confidence interval (1000 bootstraps). The dots and errorbars report the Helmholtz shear deviation (mean and standard deviation) measured in psychophysical experiments from ref. 49 (28 subjects) and ref. 48 (20 subjects). (**B**) Hering-Hillebrand deviation. Hering-Hillebrand deviation inferred by the obtained disparity distribution (solid lines) for the subjects’ (left) and random (right) fixations. The squares and errorbars report the Hering-Hillebrand deviation (mean and standard deviation) measured from psychophysical measurements of retinal corresponding points from ref. 43 (3 subjects). (**C**) Receptive field size *versus* retinal eccentricity. Mean and standard deviation (solid lines and errorbars) of receptive field size (*y*-axis) with respect to retinal eccentricity (*x*-axis), inferred by the obtained disparity distribution, among the different subjects (red) and random bootstraps (blue). The background contour map reports the population receptive field size measured from ref. 63 (12 subjects).

Differently from previous studies that directly measured the retinal correspondence with psychophysical tests^43^,^46^,^48^,^49^, here we inferred the pattern of retinal corresponding points that would be adapted to the obtained disparity statistics, and specifically to its median value (see Fig. 3A,B, and also Supplementary Fig. S1). Figure 4A,B represents the retinal corresponding points along the vertical (A) and the horizontal (B) meridian of the visual field. The figure shows the mean and standard deviation (solid lines and colored regions) computed among the involved subjects (left) or 1000 bootstraps from random fixations (right), in a direct comparison with the two deviations reported in psychophysics (circles/squares and error-bars)^43^,^46^,^48^,^49^. Table 1 also reports a numerical comparison with those data. Along the vertical meridian of the visual field, our statistics provide shear angles compliant with the physiological values of Helmholtz shear deviation (see Table 1), measured in humans (*cf.* refs 48,49). More interestingly, the retinotopic shape of shear deviation measured on humans is better explained by subjects’ fixations than by random fixation. Random fixations result in linear deviations with a “X” cross shape (see Fig. 4A, right), without significant difference between the foveal ([−1.6°, 1.6°] of eccentricity) and the perifoveal ([−6°, 6°] of eccentricity) area, as predicted by the geometry of binocular vision^12^,^13^,^25^,^47^,^50^. Conversely, subjects’ fixations are likely to account for the non-linearity of the empirical shear deviation measured in humans^46^,^48^,^49^ (Fig. 4A, left). Active fixation strategy causes the deviation to be approximately zero in a small range around the fovea (≈1°), and to increase rapidly beyond this range. The resulting effect is that the cross is transformed into an “hourglass” shape, as can also be inferred by psychophysical data^46^,^48^,^49^. Accordingly, shear angle computed in the foveal area are significantly lower than the angle computed in the perifoveal area (see Table 1).

Along the horizontal retinal meridian, our disparity statistics predict uncrossed corresponding points to the left and to the right of the fovea (see Fig. 4B), in accordance with human measurements of the Hering-Hillebrand deviation^10^,^12^,^13^,^43^,^44^,^46^. Again, subjects’ fixations are more effective in explaining the Hering-Hillebrand deviation measured in humans (see Table 1). In random fixations, the lateral shift increases smoothly with retinal eccentricity showing a convex shape. Conversely, in subjects’ fixations the deviation is almost zero within a larger foveal range (≈±1°), which rapidly increase to larger uncrossed disparities outside this range. More interestingly, in ref. 43 the deviation parameter *H* has been shown to be considerably higher in a foveal area ([−1.6°, 1.6°]), with respect to a perifoveal area of the field of view ([−6°, 6°]). This effect assesses for a significant difference between the central and peripheral visual field. In parallel to the shear angle, random fixations provide a parameter *H* which is lower than what has been measured in humans, and is almost constant over the field of view. Conversely, subjects’ fixation are much more effective in explaining the dependence of *H* on retinal eccentricity (see Table 1).

#### The Shape of the Predicted Horopter

**Figure 5:**
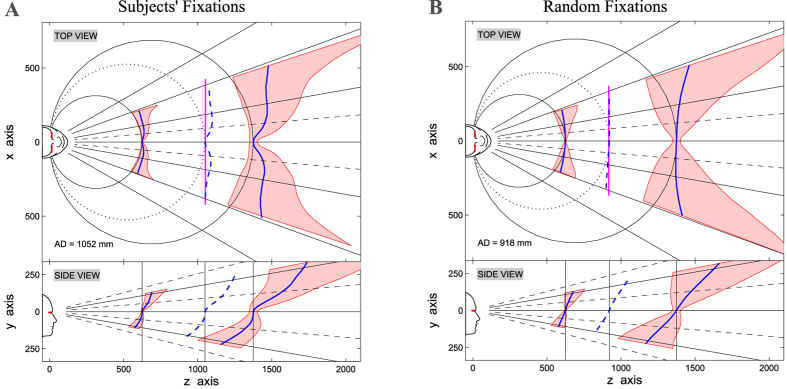
(**A**) Disparity-based prediction of 3D horopter and Panum’s fusional area for subjects’ fixations. The disparity statistics obtained by subjects’ fixations were used to infer a plausible shape for the vertical and horizontal horopter, as well as for Panum’s areas (top and side view). The horopter (blue lines) is computed as the surfaces that projects with minimum error to the pairs of predicted corresponding points. Similarly, the near and far limits of Panum’s area (red regions) were computed as the 15th and the 85th percentile of the disparity distribution. The observer is fixating straight-ahead, with a vergence angle of 7° (≈500 mm). The top view shows for reference the isovergence (Vieth-Müller) circles for the considered vergence angles, and the isoversion lines (gray lines). The dashed blue lines represent the predicted horopters computed at abathic distances, *i.e.* 3.25° vergence angle (≈1052 mm), with its the best linear fit (magenta line) and the related Vieth-Müller circle (gray dotted line). For a representation of the corresponding 3D horopter, see Supplementary Fig. S3. (**B**) Disparity-based prediction of 3D horopter and Panum’s fusional area for random fixations. The disparity statistics resulting from random fixations where used to infer the vertical and horizontal horopters and Panum’s area, as in Panel A. The abathic distance corresponds to 3.7° vergence angle (≈918 mm).

The patterns of predicted corresponding points were then used to derive the 3D optimal surface for depth perception^10^ (see Methods). Figure 5 represents the orthogonal views of the horizontal (top) and vertical (bottom) horopter (blue line) for subjects’ (left) and random fixations (right) (see also Supplementary Fig. S1), while the predicted 3D surface of the horopter is displayed in Supplementary Fig. S3. The shape we predict for the horopter agrees with the common findings empirically measured in psychophysical experiments: the viewing distance yields the expected changes of concavity and tilt on the horizontal and vertical parts of the horopter, respectively (see Fig. 5).

More specifically, whereas random fixations in near viewing result in a horopter with a smooth convex shape, when considering subjects’ fixations, a very peculiar feature arises in the very central visual field. The predicted horopter presents a pronounced *bump* within ≈1° of eccentricity that stands out of the horopter surface, and is more clearly visible considering the horizontal and vertical meridians (see Fig. 5 and Supplementary Fig. S3).

The bump at fixation provides a slight but significant deviation from the flat convex shape predicted by the theory^11^,^25^, and commonly shown in psychophysical experiments (*e.g.* see refs 10,12,13,43,44,46). Indeed, only few authors showed such an effect. A seminal study of binocular vision^51^ evidenced an everted shape on the horizontal horopter about zero eccentricity. Similar results were shown by refs 43,45, where two hypotheses were presented: the *dimple* horopter or the *flat* horopter hypothesis. In particular, the different values of the parameter *H* between the central and the peripheral field of view^43^, clearly suggest the presence of a central bump in the horizontal horopter evidenced by our statistics. With respect to similar studies that do not show the phenomenon, it is worth considering that this central bump is characterized by a small extent (less than 2°^45^), thus requiring a fine tiling of the visual space to be evidenced (*e.g.* ≈20 arcmin^43^). Similar considerations can be done for the Helmholtz shear deviation. The fact that the corresponding vertical meridians are not straight lines, results again in a bump of the vertical horopter in correspondence of the central visual field. Still, this can be ascribed to the central bias towards zero disparity, surrounded by peripheral negative disparities.

Our results assess for the hypothesis that the pattern of retinal corresponding points accounts for the first moment of disparity distribution, specifically at each retinal location. The fact that our predictions, derived by subjects’ actual fixations more closely relate to psychophysical data than those obtained from random fixations, suggests that retinal corresponding points patterns likely depend on the active fixation strategy. The predicted shape of the horopter shall be adaptive for perceiving convex, slanted surfaces in near viewing^10^,^14^,^49^, rather than to the ground plane^11^,^13^,^42^. Moreover, the bump in the central visual field clearly indicates a behavioral preference of the visual system for a *near* object surrounded by a *far* background.

While up to this point we have focused on the first-order redundancy of disparity information, in the following we will extend the analysis to the second-order regularity, *i.e.* its variability. Their relation with the disparity search zone will be analyzed with respect to the computational requirements of V1 area and the extent of Panum’s fusional area.

### Cortical Magnification Factor and Panum’s Area

While the horopter identifies the surface of optimal depth perception, stereoscopic vision is required to be effective in a wider portion of space. The Panum’s fusional area defines the region of 3D space, arranged about the horopter, where the visual system is able to integrate the visual information from the two eyes into a single percept, in order to obtain accurate stereopsis^25^,^44^,^52^. The extent of Panum’s area increases with eccentricity^25^,^53^,^54^,^55^, according to the space variant nature of the visual system, which is characterized by a finer processing of the central visual field than the peripheral one. From this perspective, it is worth considering that the growth of the average V1 receptive field size follows a logarithmic law with respect to retinal eccentricity^56^,^57^, known as the retino-cortical magnification factor^58^,^59^.

From a binocular point of view, some studies hypothesized a correlation between the magnification factor, and the extent of Panum’s area (see refs 25,54,55, as review). In fact, since accurate stereoscopic depth perception is possible exclusively within a defined range of retinal disparity^25^,^44^,^52^, Panum’s area can be seen as the phenomenological expression of the structural limits of the underlying neural mechanism^55^.

#### Receptive Field Size

The obtained retinotopic pattern of disparity variability (see Fig. 3B) was exploited to predict the receptive field size required at each retinal location (see Methods). To this aim, we exploited the *size-disparity* correlation hypothesis^25^,^60^, which predicts that with the phased-based energy model of V1 complex cells^1^,^2^,^61^, the receptive field size of the required computational resources should be proportional to the range of disparities to be encoded, for an effective representation of disparity information. On this basis, the retinotopic patterns of disparity variability (see Fig. 3A,B, bottom, and also Supplementary Fig. S1) were used to derive a model of the average receptive field sizes required to cover, at each retinal location, the range of disparities experienced by the observer (see Methods). According to neurophysiological data^62^, we considered a minimum receptive field size of 15 arcmin. The results were compared with the population receptive field size measured in human V1 area by fMRI study, courteously made available by ref. 63, since it likely reflects the range of disparities that can be encoded across the population at each retinal location.

Figure 4C shows the mean (solid line) and standard deviation (error bars) receptive field size against retinal eccentricity, as predicted by the retinal disparity distribution for subjects’ (red) and random (blue) fixations. The background contour map shows the population receptive field size measured in V1 area^63^. Table 1 reports the *rmse* computed between human data and our predictions. Both the retinal distribution obtained by random and subjects’ fixations qualitatively explain the neurophysiological data (see Table 1). Nevertheless, random fixations provide an approximately linear increase of the receptive field size, which is not consistent with the logarithmic law (see refs 25,55, 56, 57,59). Interestingly, the subjects’ data show a finer accuracy in predicting the trend of the increasing size, particularly beyond 3° of eccentricity, where data from random fixations diverge from human data.

#### Size and Shape of Panum’s Area

Qualitative representation of the associated Panum’s area was computed (see Methods). This provided a 3D shape (see Fig. 5 and Supplementary Fig. S3) that is qualitatively coherent with psychophysical measurements^48^,^50^,^54^. Considering the horizontal meridian, its extent increases with eccentricity, and shrinks in the central visual field, and a similar behavior is evident also along the vertical meridian.

In summary, our results assess an ecological correspondence between the second-order regularity of the retinal disparities experienced in peripersonal space and the variation with retinal eccentricity of the properties of binocular cortical receptive fields. Again, the results obtained with subjects’ fixation more closely account for experimental data. This suggests that active fixation strategy might have a direct role on the detectable disparity range, as well as on the underlying neural mechanisms.

## Discussion

Our results evidence a tight relation between the statistical properties of binocular disparity in peripersonal space and the neurophysiological and perceptual findings about the functional properties of the binocular visual system. The computational resources in V1 area, as the neural front-end of binocular vision in the hierarchy of the visual system, exhibit a retinotopical organization that resembles the disparity distribution experienced in near space. This likely provides an efficient representation of disparity information, usable to subsequent visual areas. Firstly, the binocular retinal correspondences likely adapt to the first-order regularity of disparity distribution, in order to recenter the search zone for stereo correspondence about the ecological range of disparities. Secondly, the increase of receptive field size with eccentricity, predicted by cortical magnification, conforms to the second-order regularity to allow an efficient coding of binocular visual information with a limited number of resources. To this purpose, the binocular neural mechanisms are likely to embed, at a structural level, the natural disparities experienced by a fixating observer, rather than those obtained from random fixation strategies. The behavioural preference for object-oriented fixations results in a significant difference of binocular processing between the central and the peripheral part of the visual field, which we will now discuss in comparison to other functional aspects of binocular vision.

### Comparison with Other Studies of Disparity Statistics and Limitations of the Present Study

Two complementary factors define what is the pattern of disparity occurring on the retinas: the 3D structure of the visual environments and where the eyes are gazed within it. In previous studies, 3D models of scenes, either synthetic^18^,^19^ or natural^16^,^20^, satisfying the first factor, but relied on simulated eye movements. In order to gather realistic disparity statistics, it is worth considering that actual human fixations of a subject are neither randomly nor evenly distributed within the 3D environment^21^,^22^,^23^. Conversely, the natural environment is an ideal “setup” to collect disparity statistics of a behaving observer performing everyday tasks^14^. The subject is free of moving and his behavior is natural, so as his fixation within the visual environment. Whereas this approach has the advantage of resorting on actual human eye movements, it comes to the price of a less precise knowledge of the structure of the environment, since the 3D scene has to be estimated, introducing inevitable measurement errors, particularly in critical areas, like depth edges.

The methodology we propose lies in between of these two approaches. The accurate 3D models of natural scenes that we use provide a naturalistic and contextually rich visual stimulation, together with the required degree of control over the key variables, namely the ground-truth structure of the scene and the eye movements performed while exploring it. Beside a general agreement with the data generally presented in disparity statistics studies, our methodology allowed us to evidence a subtle but significant difference between the central and the peripheral visual field. In our opinion, we were able to evidence this phenomenon thanks to the specific characteristics of the experimental setup adopted. Exploiting 3D models of the environment with less than 1 mm accuracy, the obtained ground-truth disparity have high accuracy and precision, particularly in correspondence of depth edges, which is where the algorithms for depth estimation mostly fail. Without the need of estimating the 3D structure of the environment, the calibration procedure simply relies on a robust method provided by the eye tracker producer, and its precision is enhanced by stabilizing the subject’s head with a chin rest. From this perspective, our setup allows for accurate and precise statistics of retinal disparity, particularly where disparity is small, *i.e.* in the foveal area, allowing us to evidence a bumped shape in the horopter, instead of a flat one.

Nevertheless, the high degree of control of the proposed setup, required for accurate ground-truth disparity, comes at the price of some limitation with respect to more natural setups: the considered scene are virtual reality versions of natural environments experienced through a screen with limited field of view, and subjects’ head is stabilized in a chin rest, preventing normal head movements, which are quite natural during visual exploration. Moreover, the virtual scenes we used have a recurrent 3D structure, since they are composed by discrete objects against a desktop. To some extent, the central bias we evidence might depend on the task performed by the subject in these scenes. Nevertheless, it is intuitive how similar disparity patterns are likely experienced during actions performed in close viewing, like social interaction (*i.e.* where gaze is directed onto faces), or manipulating actions, (*i.e.* with gaze directed onto the own hand or the grasped object). This allows us, to some extent, to generalize the results to more active and variable everyday visual experiences, as those performed in ref. 14.

### The Shape of the Horopter: the Flat vs the Dimple Hypothesis

Our results are generally consistent with the findings presented in literature, for what concerns both subjects’ and random fixations. The foveal bump, present in subjects’ fixations only, thus derives directly by the fixation strategy performed by human observers. To the best of our knowledge, this effect has seldom been evidenced or hypothesized in the literature, and it has never been related to fixation strategy. In seminal work on stereopsis^51^, the measured shape of the horizontal empirical horopter clearly shows a bump at fixation. The author argues that theoretical and empirical shapes considerably differ, but no possible explanation about the bump is provided. The first overt hypothesis of this effect can be found in ref. 45. The authors proposed two different theories about the horopter’s shape, to account for an adaptation mechanism of retinal correspondence that facilitates fusion across a range of viewing distances. A *global* shift provides a flattening of the horopter, whereas a *local* shift yields a dimple in the central field of view (≈2°). Subsequent studies demonstrated how retinal correspondence is fixed^43^,^49^, neglecting the possible adaptation. Even if the bump can also be evinced in data from ref. 43 (see Fig. 4B), no light has been shed on the actual shape of the empirical horopter. To our opinion, the complexity in evidencing this effect originates by the required resolution. Retinal correspondence studies, investigating a wide field of view, commonly used one or two degrees resolution^10^,^46^,^48^,^49^), which is barely sufficient to reveal the bump, since it likely concerns the first one/two degrees of visual field. Only two experiments have been performed with finer resolution, especially in a foveal area^43^,^51^. From this manuscript onward, the general characteristics of the horopter’s shape were no more discussed or questioned, and all the studies basically confirmed them^14^,^46^,^48^,^49^. According to our analysis, the presence of a bump at fixation can be evinced by psychophysical data, for both the Helmholtz shear deviation (see Fig. 4A)^46^,^48^,^49^ and the Hering-Hillebrand deviation (see Fig. 4B)^10^,^43^,^46^,^51^. Data from active fixation strategy allows for a more precise and compliant explanation of psychophysical and neurophysiological data (see Table 1), strongly supporting this hypothesis.

As a matter of fact, our results provide a formal reinterpretation of the shape of the empirical horopter. The similarities between disparity statistics we obtained from random and subjects’ fixations, suggests that the tuning of the visual system partially derives from the structure of natural environments and by the attitude in coping with the environment itself. The resulting 3D shape of the horopter has almost constant convexity and is tilted top-down, resembling the average predominant environmental surfaces, as the ground plane^10^,^11^,^12^,^13^,^50^ or planar objects in near viewing^10^,^14^. Particularly at large eccentricities, the horopter’s shape is equivalent in both cases, showing how the structure of the environment overshadows any possible effect from fixation strategy. This peculiar feature evidenced in subjects’ data, derives as a direct consequence of active fixation strategy, warping the horopter at fixation. In fact, if we manually select those fixations that are gazed on objects among the random dataset, qualitatively equivalent results can be obtained. This clarifies that the bump at fixation “flattens” due to the fixations that are not centered on objects, consequently predicting a smooth 3D shape for the empirical horopter. The resulting shape finely resembles a behavioural 3D structure that is frequently experienced by an observer, *i.e.* a close object out of a far background.

### Sensory and Motor Fusion

Normal binocular vision requires an effective cooperation between sensory fusion (stereopsis) and motor fusion (vergence). Despite the large agreement on the general principles that regulate binocular vision, a significant intra-subject variability is likewise evident. Just to mention a few: fixation disparity has been measured in near viewing in the range of −30 and 120 arcmin^64^; ocular torsion is proportional to vergence angle by a parameter between 0.20 and 0.41^28^; the empirical corresponding points show different patterns from subject to subject^48^,^49^.

The present work relies on two assumptions: 1) the optical axes are perfectly aligned on the object of interest, and 2) the eye torsional alignment respects the binocular extension of the Listings Law^27^,^28^,^29^. In real conditions, the range of disparity supported in a foveal area has two meanings. First, it should account for fixation disparity^64^, and second, it should allow for an effective vergence control^14^. Some functional and computational advantages can be obtained by fixation disparity. Assuming a positive fixation disparity, as commonly measured in normal subjects (*e.g.* 40 arcmin^64^), the distance between the predicted and the geometric horopter decreases (see animated Supplementary Fig. S4). Accordingly the *global* disparity experienced by the subject is reduced, providing a computational advantage and a greater fusional vergence response, with respect to a configuration where the optical axes are perfectly aligned at fixation^45^,^65^. Besides, a *local* adaptation of the retinal correspondence accounts for a null perceived disparity at fixation, *i.e.* the central bump. Hence, fixation disparity likely produces or results from a sensory adaptation to a small motor ocular misalignment^66^, assessing the interplay between motor and sensory fusion.

From this perspective, normal binocular vision is more likely to occur on the basis of an effective interplay between the two fusional mechanisms, rather than on a “perfect” functionality of the single components separately. Since stereopsis and vergence have been shown to share the same computational substrate^67^,^68^, this suggests that the general functionality is due to the structural properties of the binocular visual system. Besides, specific intra-subject variability might reflect different strategies implemented by the visual system, deriving from the personal development of the subject and its own individual visual experience^69^.

### Development of Binocular Vision

Cortical visual functions in humans are very immature at birth, and visual experience plays a key role in the development of the neural system underlying visual perception (see refs 7, 8, 9, but also^69^. The first two/three postnatal months are necessary to develop basics monocular aspects of visual perception, like visual acuity, contrast sensitivity and orientation selectivity^70^. Stereopsis emerges later between the 3rd and 6th month^71^,^72^. Regarding the motor side, a rough binocular alignment has been shown to be already present at 1 month of age, thus anticipating the onset of the sensory side of binocular vision^73^.

While the relation between the 3D environment and the geometry of visual system is by itself sufficient for a qualitative prediction of the functional properties of the binocular visual system^11^,^15^, considering the subjects’ actual exploratory fixations suggests some observation alongside the development of the visual system. In fact, a sensitivity to crossed disparities is already present at about 3 months of age, slightly before the sensitivity to uncrossed disparities^74^. From a developmental point of view, this preference can be explained by the fact that *early* bottom-up visual features (like motion, luminance contrast, color, etc.) have a prominent role in guiding the gaze of the infant^75^. As a behavioral explanation, the infant attention is firstly attracted by a near stimulus (like the parent’s face or a toy) surrounded by a far environment. The behavioral preference for close locations^21^,^22^,^23^ and objects^24^, is thus reflected by a preference of the visual system for crossed disparities. In fact, a close/crossed figure surrounded by a far/uncrossed ground facilitates the visual system at different levels: it is effective in providing a better recognition of disparity-defined shapes^76^ and perceptual facilitation^77^, as well as in better stimulating higher visual areas related to figure-ground segregation and object recognition^3^,^4^,^78^. While this selectivity to a disparity-defined convexity is likely to represent the binocular aspect of the figure-ground segregation process^4^, our results suggest how this mechanism is likely to lay his grounds already in the early stages of the binocular visual system.

## Conclusion

The presented results suggest the role of active visual behavior in conditioning the visual neural system to the structural characteristics of the visual environment. As direct consequence, fixation strategy provides a behavioral influence also on the binocular aspects of the developing visual system. Since fixation strategy is primarily committed to tasks such as object recognition and manipulation, the neural substrate underlying binocular vision shall specifically embed this preference. The bump at central fixation suggests how the binocular visual system is not simply tuned to a convex and slanted surfaces in near viewing^10^,^14^,^49^, but also to a disparity-defined convexity compliant with a near object of interest surrounded by a far background, as the binocular aspect of the figure-ground segregation process. The obtained results, being derived by disparity statistics collected in near viewing, only, nurture the significance of peripersonal space in tuning the binocular functionality of the visual system. To which extent this tuning might be due to developmental or evolutionary reasons, requires a further and accurate research.

## Materials and Methods

### Methodological Approach

In this paper, we implemented an experimental setup that allows computing accurate and precise *ground-truth* disparity, corresponding to actual subjects’ fixations in a virtual 3D world. The proposed approach relies on three complementary parts: 1) 3D virtual models of natural scenes in peripersonal space, 2) a stereoscopic experimental setup, and 3) a stereoscopic virtual reality simulator. The 3D virtual models have been obtained by a high accuracy 3D laser scanner, and consist of accurate depth information and natural texture. The conceived experimental setup, combining binocular eye tracking with 3D stereoscopic display, allows us to measure the active fixations of human subjects while exploring the 3D scenes. The virtual reality simulator has been implemented to achieve two distinct objectives: 1) to obtain stereoscopic visual stimuli to be displayed to subjects within the stereoscopic setup, and 2) to compute the ground-truth disparity patterns associated to the measured subjects’ actual fixations.

The subject is positioned on a chin-rest in front of the 3D screen, while a 3D visual stimulus is displayed. Since the position of the subject with respect to the stereoscopic screen and the virtual scene is known, the eyetracker data were used to reconstruct the position of subject’s gaze within the 3D scene. Nevertheless, the high degree of control of the implemented setup comes to the price of some limitation in the naturalness of the presented stimuli. The subject’s head is stabilized by the chin rest, the field of view is constrained by the screen size, and possible conflicting cues may affect the realism of the stimuli. From this perspective, we carefully designed and integrated the three modules composing our system, in order to relax those limitations:

- The vergence-accommodation conflict, which produces visual fatigue and discomfort, is mitigated designing the stereoscopic setup geometry^79^. The focal distance of the display matches the simulated distance of the virtual scene^80^. The large screen size allows us to place it far from the observer (115 cm), reducing the effect of focus conflict^80^, while respecting the 3D comfort zone of vergence-accommodation conflict^79^,^81^;
- The large screen mitigates the framing effect, since it provides a sufficient large field of view (≈44° horizontally and ≈24° vertically), that allows horizontal and vertical gaze movements in a range close to that measured in psychophysical experiments during real world exploration^14^,^82^,^83^;
- The stereo pair is computed using an *off-axis* technique with skewed *frusta*^84^, providing geometrically correct depth cues for a single 3D screen, namely perspective, binocular disparity and occlusions^79^,^85^;
- The stabilization of subject head by a chin rest prevents conflicting parallax motion^84^;
- The scene lighting has been handled by the Coin 3D library to obtain realistic shading effect.

Accordingly, the proposed approach aims to make existing conflicts less obvious, so to provide a more natural and less fatiguing visual stimulation.

Next, we computed the retinal disparity patterns associated to the subjects’ actual fixations measured during the visual exploration task. Notably, the proposed setup allows us to obtain the *ground-truth* disparity falling on the retinas. In fact, the virtual reality simulator was specifically implemented modifying the *toe-it* technique to mimic the binocular eye posture of the human visual system^86^, thus considering eye convergence and cyclotorsion^27^. The proposed approach allowed us to collect statistics of binocular disparity experienced by an observer during free exploration of natural environments in near viewing. The resulting data for disparity statistics were qualitatively and quantitatively compared with the known functional properties of the binocular visual system.

### Notation and convention

The following notation is used in the paper. Horizontal disparities are represented as positive (red colors in Fig. 3) for objects closer than the fixation point, *i.e.* for crossed disparities; uncrossed disparities are negative (blue colors). The definition of the vertical component of the disparity vector is quite controversial in the literature; here we refer to the difference between the elevation longitude of the images in the two eyes^32^ which is equivalent to the vertical shift in the Cartesian coordinates on the plane on which one projects the hemisperical retina. In figures, they are represented as positive if elevation is right-hyper, *i.e.* higher in the right than in the left eye (red colors), and negative for the opposite (blue colors). The following quantities: retinal disparity, retinal eccentricity (with respect to the fovea), retinal deviations, and receptive field size are reported in degrees of visual angle. The root mean square errors (*rmse*) for the Helmholtz shear and Hering-Hillebrand deviations and the receptive field size are reported in arcmin. Subscripts R, L indicate that the quantity is referred to the left, right eyes/cameras, whereas C refers to the cyclopean eye/camera. The gaze direction is defined as azimuth (*H*), elevation (*V*) and vergence (*ν*) angles.

### 3D range and image data

In order to develop a naturalistic stimulation of the visual system, we created realistic models of *real* natural environments in peripersonal space. The models are composed of the depth map (Z-buffer) of the scenes together with the actual object textures. To this purpose, we used a 3D range laser scanner Konica Minolta Vivid 910, appropriate to create 3D virtual models in the close range. The laser scanner provides digitized images with accurate range data (±0.1 mm) at close ranges (from 0.6 *m* to 1.2 *m*), with high spatial resolution (up to 307,200 points per acquisition). The device is also endowed with an RGB camera that acquires the actual image texture (640 × 480 pixels), aligned to the 3D scene. The scanner has a variable field of view that can be adjusted with three interchangeable lenses (focal distance: TELE 25 mm, MIDDLE 14 mm and WIDE 8 mm), in order to accommodate the measurement of objects with various sizes and distances from the lens.

The virtual models were created, according to the following procedure. The scenes were composed of real-world objects arranged in a cluttered way in order to recreate every day life situations in a high complexity structure. A first scene recreates a kitchen table, while a second scene recreates an office desk (see Fig. 1A). The single objects were first acquired with the laser scanner in TELE modality, to obtain high spatial resolution models. Each object was scanned from different positions to allow a full 360° acquisition, in order to minimize the occlusion problems that occur when one simulates changes in the vantage point of the virtual observer. Next, we took eight scans of the entire scenes, rotating around it by step of 45°, in order to have a 360° coverage. The acquired views were then registered to create a raw model of the scene. Finally, the single object models were aligned within the raw model of the scene, recreating the original scene as a composition of high resolution and high precision object models.

The final result is a virtual model of the selected environments characterized by high resolution (more than 13,000,000 of points) and accuracy (≈0.1 mm), as well as by the actual objects’ textures (see Fig. 1A).

### Virtual reality simulator to model visual systems

Once obtained the virtual models of our 3D environments, a virtual simulator is necessary to compute the stereoscopic image and the associated ground-truth disparity patterns.

The common approach in virtual reality is to configure the virtual cameras with parallel optical axes^87^. In such a configuration, the resulting disparity patterns are characterized by zero vertical component, and the horizontal one is exclusively uncrossed, *i.e.* corresponding to objects farther than the screen plane (*e.g.* see ref. 87). A vergent geometry resembling the human fixation posture, is seldom considered^16^,^88^. As we want to consider natural eye postures, it is necessary to obtain realistic disparity patterns that resemble those experienced by human subjects in real conditions^14^,^88^,^89^.

To this purpose, we implemented a virtual simulator that satisfies the requirements imposed by an active vision system. The virtual reality tool simulates the vergence movements of two cameras on a point in space^86^, allowing us to compute the horizontal and vertical ground-truth disparity maps. The software is based on a C++/OpenGL architecture and on the Coin3D graphic toolkit (www.coin3D.org). The Z-buffers of the virtual models are used for the 3D rendering of the scenes. This allowed us to compute, beyond the images projected on the left and right cameras, the corresponding ground-truth disparity maps.

The disparity patterns resulting by the vergence posture are characterized both by uncrossed and crossed disparities for the horizontal component, and the vertical component is equal to zero along the horizontal and vertical meridians, only^38^. To the purpose of this study, we endowed the virtual reality tool with the possibility to simulate the viewing posture of the cameras following a cyclo-torsional alignment, which implements the binocular extension of Listing’s Law^27^. Hence, if the gaze direction is different from straight-ahead, the disparity patterns also depend on the relative cyclotorsion between the two eyes^27^.

#### Computation of the stereo image pairs

**Figure 6:**
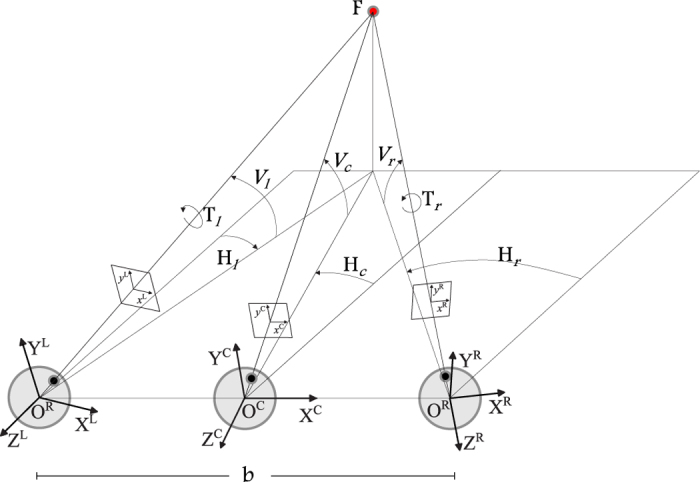
Geometrical sketch of the*toe-in* technique. The left and right camera frames: (*X*^*L*^, *Y*^*L*^, *Z*^*L*^) and (*X*^*R*^, *Y*^*R*^, *Z*^*R*^). The left and right image planes: (*x*^*L*^, *y*^*L*^) and (*x*^*R*^, *y*^*R*^). The camera optical axes are directed towards the fixation point F. b represents the baseline, i.e. the interocular distance. (*H, V, T*)_*L*/*R*_ represents the azimuth, elevation and torsion angles for the left and right cameras, respectively.

In order to compute the stereo pairs corresponding to two cameras in a verging posture, we used the *toe-in* method: each camera is directed to a single fixation point through a proper rotation. The geometrical sketch of the optical setup of an active stereo system and of the related *toe-in* technique is shown in Fig. 6. The relation between the 3D world coordinates **X** = (*X, Y, Z*) and the homogeneous image coordinates **x** = (*x, y*, 1) is described by a general perspective projection model: **x** = **X**/*Z*.

In general, the disparity map of a stereo pair is calculated with respect to the image plane of the left or of the right image^87^. To avoid asymmetry problems we referred to the image plane of a cyclopic camera^90^, located in the mid point between the left and right cameras, pointing along the gaze line at the selected fixation point. Given the projection of a 3D virtual point on the cyclopic image plane, the disparity maps were computed by the correspondent projections in the left and right image planes.

Starting from the image coordinate **x**^*C*^ of the cyclopic image and the depth values *λ*^*C*^ obtained by the Z-buffer, the image coordinate **x**^*L*^ and **x**^*R*^ of the left and right view are obtained in the following way:


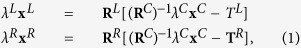


where **R**^*L*/*R*/*C*^ are the rotation matrices defined by the actual fixation point respectively for the left, the right and the cyclopic camera in the head fixed reference frame, and **T**^*L*/*R*^ are the positions of the left and right camera with respect to the cyclopic one, in the head reference frame. Finally, the horizontal disparity *d*_*x*_ = *x*^*R*^ − *x*^*L*^ and the vertical disparity *d*_*y*_ = *y*^*R*^ − *y*^*L*^ are computed.

#### Cyclo-torsion of the eyes: Binocular Listing’s Law

The actual positions of the human eyes are restricted in such a way that there is only one eye position for every gaze direction. The eyes obey to constraints that relate the amount of torsional angles for each gaze direction to the vergence angle, according to the Listing’s Law and its binocular extension^27^,^28^,^29^. Accordingly, each eye assumes only those orientations that can be reached from primary position (*i.e.* gaze straight ahead) by a single rotation about an axis lying on the Listing’s plane. This plane is orthogonal to the line of sight when the eye is in the primary position^29^. Moreover, the torsional posture of each eye changes when the eyes converge on a near object, following the binocular extension of the Listing’s law^28^. During convergence, the Listing planes rotate temporally and roughly symmetrically by *φ*_*l*_ and *φ*_*r*_ angle, for the left and the right eye, respectively. The more convergence exists, the more the plane rotates temporally, implying that during convergence, there is a relative excyclotorsion on upgaze, and a relative incyclotorsion on downgaze. The resulting posture provides an alignment of the horizontal meridians of the left and right eyes. This alignment reduces the search zone for retinal correspondence, thus providing a perceptual optimization for stereopsis^10^,^42^,^91^.

On the top of these considerations, we derived the formulation^92^ required to obtain a camera posture that mimics natural eye posture, also taking into account the tilting of the Listing’s plane:


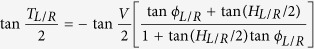


with


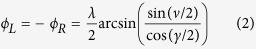


where *ν* = *H*_*R*_ − *H*_*L*_ and 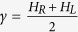 are the vergence and the version angles, *T*_*L*/*R*_ is the cyclorotation, and *H*_*L*/*R*_ and horizontal angle of left and right eyes, whereas *V* is their common vertical angle (see Fig. 6). The parameter *λ* controls the balance between the motor advantage of Listing law and the perceptual optimization for stereopsis. In all the following simulations we decide to adopt *λ* = 0.8^14^,^29^,^42^.

### Experimental Procedure

The virtual reality simulator was used to obtain realistic stereo pairs to be presented dichoptically to the subjects for visual exploration tasks in the 3D scenes. Four subjects were included in the experiment, two males and two females, 25 to 34 years old. The human subjects protocol was conducted ethically according to the principles line of the Declaration of Helsinki, and was approved by the Ethics Committee of San Martino Hospital (Genova, Italy, Protocol Number P.R. 400REG2015). All of the participants voluntarily enrolled in the study and signed an informed consent before we began the experiment. The subjects, all with normal vision and normal stereoacuity, were naive to eye tracking. The subjects were also unaware of the goal of the experiment, and were instructed to freely explore the scenes.

#### Experimental Setup

The stereo pairs were displayed on a 42-inch (930 × 523 mm) LG 42LW450A stereoscopic LCD screen. The screen had a resolution of 1080 × 1920 pixels, with a refresh rate of 100 Hz. Stereoscopy was obtained with a pair of passive circularly polarized glasses (RealD 3D technology).

The subjects were placed at a fixed distance of 1150 mm (≈3° of vergence) from the screen, *i.e.* the distance for which the stereoscopic views were rendered. The head was stabilized through a chin rest, in order to have the subjects centered with respect to the screen. Accordingly, the sagittal plane of the subject was coincident with the vertical half of the screen, while the transverse plane passing through the eyes’ center was coincident with the horizontal half of the screen.

A SMI RED 250 mobile eye-tracker was used to record the eye movements. The accuracy of the eye-tracker declared by the manufacturer is 0.4 degree. The eye tracker was placed on a stand at ≈600 mm from the observer, in the lower part of the subject’s field of view, in order not to occlude the screen. First, a calibration was executed and validated by exploiting the procedures provided by the manufacturer. The procedure was extended in order to mitigate possible errors due to fixation disparity^64^,^93^. Each eye was calibrated separately, in order to increase the accuracy of the measured eye position in binocular tasks (see ref. 94).

#### Visual Stimuli

The visual stimuli consisted in twenty scenes obtained by the two virtual models, ten per each model, according to the following procedure. For each model, the virtual head was positioned facing the scene, at a fixed distance of 1150 mm from the closest object along the cyclopean gaze line, as in the experimental setup. The position vector of the head varied uniformly with the azimuth angle in the range [−60° 60°] by steps of 30°, for two elevation angles of 30° and 45°, thus defining 10 different vantage points for each virtual model. The simulator was thus configured to resemble the experimental setup: the camera resolution was set to the screen resolution, the field of view was set to cover the actual screen size, *i.e.* ≈44° horizontally and ≈24° vertically, and the stereo camera had an interocular distance of 60 mm. For each of the defined head position, we obtained the stereo pair to be presented to the subject (*e.g.* see Fig. 1C,D). To this purpose, it is worth considering that images generated by the *toe-in* method might be correctly displayed to a subject only using two display devices, one for each eye, aligned with the retinas. Displaying such images on a single stereoscopic monitor, would result in exaggerating the vertical disparities falling on the retinas, thus violating the correctness of the 3D perspective^85^,^95^. In order to provide the correct perspective, we projected the left and right images of the *toe-in* stereo pair onto the screen plane, interpolated the projections to the actual screen size and resolution. Formally, the obtained stereo pairs correspond to those that can be obtained considering an *off-axis* technique with skewed *frusta*^84^.

#### Procedure for Subjects’ Fixations

Each stereo pair was presented dichoptically to each subject for 18.5 seconds on the screen, while recording the binocular eye movements with the eye tracker. Each stimulus was preceded by a white cross on a uniform gray background, presented for 1.5 seconds. For each of the eye, separately, the 2D gaze points on the screen were mapped into the corresponding 3D gaze point in the virtual scene. Next, the 3D points were projected on the cyclopean image plane, according to Eq. 2. The binocular scan path was then computed as an average between the left and right points on the cyclopean image, and were used to derive a heatmap of the subjects’ actual binocular fixations (see Fig. 1C, bottom). The 2D fixation points on the cyclopean image were extracted by the binocular scan path, considering a minimum fixation time of 0.3 sec (red dots in Fig. 1C), and correspond to the “hot” points of the heatmap. The fixation points in the 3D scene (see Fig. 1C, top) were obtained by back-projecting them on the closest visible surface of the scene, and were then used to compute the gaze angles. Thus, each fixation is described by the position and orientation of the head in the world reference frame, together with the vergence, azimuth and elevation angles of the binocular line of sight. Each subject performed a variable number of fixations for each scene, ranging from 24 to 43.

Finally, the virtual reality simulator was used to direct the binocular gaze to the targets, and to compute the left and right retinal images, together with the associated horizontal and vertical ground-truth disparity maps (see Fig. 1C, top). This procedure yielded four stereoscopic datasets, one for each subject, composed of ≈733 ± 122 fixations (mean and standard deviation among fixation number).

#### Random Fixations

The following procedure was used to simulate random fixations within the same virtual words. The gaze directions from the four subjects were gathered in a single list, composed of 2932 fixations. The vergence information was removed, while keeping the azimuth and elevation of the binocular gaze line. Next, each fixation was used in the virtual simulator to obtain the stereo pairs corresponding to that gaze direction, but from a head position different from the one were the fixation was measured (see Fig. 1D, bottom). In this new configuration, the vergence angle was set to correctly fixate on the closest object along the binocular line of sight. The procedure was repeated 5 times, obtaining a dataset of ≈15000 fixations. This procedure, randomizing the relation between the head position and the gaze direction, allowed us to obtain a disparity dataset of random fixations of a virtual observer in the same 3D scenes used for measuring subjects active fixation (see Fig. 1D, top). This approach ensures that the probability distribution of the gaze direction is within the natural variability occurring in human visual exploration^14^. A systematic data set of stereo pairs, released for public use, can be found at: www.dropbox.com/s/w97jl5ea4utsvaf/3D_DATASET_EXAMPLE.zip?dl=0.

### Statistical data analysis and interpretation

For a statistical comparison with subjects’ fixations, random fixations of a virtual observer were simulated on the whole dataset of 15000 images, exploiting a bootstrapping technique with 1000 reduced datasets, each consisting of 733 samples, *i.e.* the mean size of the subjects’ datasets. We computed the disparity distribution considering the central field of view, and on selected retinal areas^14^, *i.e.* differentiating between the upper and lower hemifields for the horizontal disparity, and between crossed quadrants for the vertical disparities (see Fig. 2A). More particularly, a retinotopic representation of the disparity distributions (see Fig. 2B) evidenced possible systematic relations between the characteristics of the distribution and the corresponding retinal location. Aiming to obtain a parametric representation of the disparity statistics, it is worth considering that the distributions are not symmetric about the mean value. Whereas for normal distributions the mean and standard deviation are representative quantities of the central value and variability of the distribution, in our case we adopted as first moments the median and the standard deviation, to describe the main features of the distribution^96^. The first moment has been computed for the horizontal and vertical disparity, separately, while the second moment has been computed along the principal orientation of the vector disparity. The features were then represented as retinotopic patterns (see Fig. 3A,B).

In order to assess if the random and subjects’ fixations have similar statistical properties, we computed the *χ*^2^ probability (*p*-value) of the Mahalanobis distance^97^ between the subjects’ disparity distribution and the random distribution computed at each bootstrap. This methodology was adopted because it is able to generalize beyond the set of normal distributions^97^. The computation was performed for horizontal and vertical disparities, separately (see Fig. 3C).

#### Retinal Corresponding Points Prediction

Since retinal correspondence is considered to adapt to the disparity statistics naturally occurring on retinas^10^,^14^,^42^,^43^,^49^, we inferred a plausible pattern of retinal corresponding points that would be adapted to our statistics. The median disparity pattern (Fig. 3) was thus used as the value of disparity to be removed at each retinal location. This approach was followed along the vertical (see Fig. 4A) and horizontal (see Fig. 4B) meridians of the field of view, respectively, as well as over the central part of the field of view (see Supplementary Fig. S2). The resulting Helmholtz shear and Hering-Hillebrand deviations were then compared with data from psychophysical measurements of the same quantities. For the Helmholtz shear deviation, data were aggregated from^48^,^49^, for a total of 48 subjects, and were represented as mean (circles) and standard deviation (error-bars) (see Fig. 4A). The amount of shear is commonly represented by the angle between the corresponding vertical meridians of the eyes^48^,^49^. We used a quadratic fitting to represent the predicted corresponding points along the left and right vertical meridians and to compute the shear angles at the intersection. Table 1 also reports the shear angles, obtained on subjects’ and random fixations, as well as data form psychophysics^48^,^49^, for a direct comparison.

For the Hering-Hillebrand deviation, psychophysical data were collected from ref. 43, where the authors measured the retinal deviation corrected for the fixation disparity measured at different viewing distances on three subjects. The data were represented as mean (squares) and standard deviation (error-bars) (see Fig. 4B). The distance between the predicted patterns of retinal corresponding points and the mean deviation measured in humans, were computed as root mean square error (rmse) and reported in Table 1. A quantification of the Hering-Hillebrand deviation has been originally introduced by ref. 44, who defined a deviation parameter *H* based on relative horizontal disparities in the left and the right eyes, *i.e.* the perceived visual direction *α*_*L*_ and *α*_*R*_, and a factor *r* describing the magnification in the right eye relative to the left: *H* = cotan(*α*_*L*_) − *r*cotan(*α*_*R*_). The formula can be elaborated to make it explicit the dependence on the horizontal retinal disparity *δ*_*H*_ and refer to the eccentricity of a single eye^10^, so that:


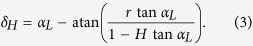


Hence, we used the retinal corresponding points along the horizontal meridian, predicted by our statistics, to minimize Eq. 3 and obtain an estimate of *H*. The magnification parameter has been set to 1, *i.e.* equal magnification in the two eyes. In order to differentiate between the central and the peripheral field of view, the computation has been performed over the first ±1.6° and over the first ±6° of retinal eccentricity^43^. Table 1 reports the values of *H*, obtained on subjects’ and random fixations, as well as data form psychophysics^43^, for a direct comparison.

#### Receptive Field Size Estimation

A phased-based energy model^1^,^2^,^61^ was postulated to derive computational implications about the early vision resources required to encode the disparity range that results from our statistics, at each retinal eccentricity. According to the model, the detectable disparity range Δ supported by a population of binocular energy neurons is:


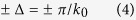


where *k*_0_ is the radial peak frequency of the receptive field, and ±*π* is the maximum applicable phase difference between the left and right receptive fields. On this basis, we used the statistical disparity ranges to compute the required size of the population receptive fields. Even if a certain degree of variability has been measured in the shape of the receptive fields^62^e sake of simplicity, we assumed a constant ratio between the radial peak frequency and the size of the receptive field. Specifically, the spatial extent *σ* of the envelope of the receptive field was obtained as:


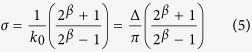


where *β* relates to the spatial-frequency bandwidth and typically varies in a limited range around 1 octave. Finally, the average receptive field size was computed as the size of the spatial support of the receptive field, set as 5*σ*.

The variability the disparity distributions (see Fig. 3, bottom) was then used to predict the receptive field size with respect to eccentricity (see Fig. 4C), for subjects’ (red) and random (blue) fixations. The prediction for each subject/bootstrap was obtained by averaging among different meridians of the field of view (mean and standard deviation). According to neurophysiological data^62^, a minimum receptive field size of 15 arcmin was considered. Our predictions have been compared with the data of population receptive field size in area V1 derived by fMRI measurements, courteously made available by ref. 63. Clearly, the fMRI measurements depend both on the size of the individual neurons’ receptive fields on their degree of overlap. The contour map in the background represents the population receptive field size aggregated from 12 human subjects. The distance of the predicted trends form the mean trend derived by human measurements was computed as root mean square error (*rmse*), and reported in Table 1.

#### Horopter and Panum’s Area Prediction

Psychophysical evidences demonstrated that when the projections of an object in space fall onto retinal corresponding points, the stereopsis is more precise^44^,^51^. Accordingly, the patterns of empirical corresponding points, measured in human subjects, have been used to infer the shape of the optimal surface for depth perception, *i.e.* the empirical horopter in the 3D space^42^.

Similarly to ref. 42, we directly exploited the patterns of retinal correspondence predicted for subjects’ and random fixations (see Fig. 4A,B, but also Supplementary Fig. S2), to compute the shape of the horopter in the 3D space for a given a gaze line direction and a distance of fixation (see Fig. 5 and Supplementary Fig. S3). For each pair of predicted corresponding points, we projected two rays originating from the eye nodal points, passing one from the point on the left retina and one from the corresponding point on the right retina. Since the predicted corresponding points derive by different gaze directions, as well as by different point in space, the two rays do not necessarily intersect. Hence, the point belonging to the horopter was computed as the point at minimum Euclidean distance between the two rays, through a Moore-Penrose pseudo-inverse method.

Similarly to the horopter, we computed the near and far limits of Panum’s area (see Fig. 5), considering that its extent should cover a range of disparity proportional to the variability of the distribution. The near and far limits of Panum’s area were computed as the 15th and the 85th percentile of the distribution, in order to take into account its asymmetry and the possible presence of outliers (see Fig. 2A). In such way, the computed Panum’s area has an extent that covers a large percentage of the retinal disparity (roughly one standard deviation of a normal distribution), and comes to be tuned to the specific characteristics of the disparity distribution experienced at each retinal location (see Fig. 5).

## Additional Information

**How to cite this article:** Gibaldi, A. *et al*. The Active Side of Stereopsis: Fixation Strategy and Adaptation to Natural Environments. *Sci. Rep.* **7**, 44800; doi: 10.1038/srep44800 (2017).

**Publisher's note:** Springer Nature remains neutral with regard to jurisdictional claims in published maps and institutional affiliations.

## Supplementary Material

Supplementary Material

## Figures and Tables

**Table 1:** Numerical comparison with psychophysical and neurophysiological data.

	Eccentricity	Shear Dev.		H-H Dev.		V1 RF Size
	[deg]	*θ[deg]*	*rmse[arcmin]*	H[deg]	*rmse*[arcmin]	*rmse*[arcmin]
Random Fix.	[−1.6, 1.6]	1.26 ± 0.12	—	0.12 ± 0.03	1.80	—
Subjects’ Fix.		0.45 ± 0.09	—	0.73 ± 0.05	1.12	—
Psycho. Data		—	—	1.51 ± 0.23	—	—
Random Fix.	[−6.0, 6.0]	1.19 ± 0.10	0.62	0.13 ± 0.06	4.73	56.40
Subjects’ Fix.		1.41 ± 0.13	0.38	0.26 ± 0.05	2.35	15.37
Psycho. Data		1.66 ± 0.88	—	0.28 ± 0.09	—	—

The Helmholtz shear deviation, Hering-Hillebrand deviation (H-H) and V1 receptive field size derived by our statistics (for random and subjects’ fixations) are reported against the data available in psychophysical and neurophysiological literature. For what concern the Helmholtz shear deviation, we compared the value of the shear angle *θ* (mean and std) with the angles measured in refs 48,49, whereas for the Hering-Hillebrand (H-H) deviation we computed the values of *H* (mean and std)^42^,^50^, for a comparison with data from ref. 43. We also reported the *rmse* between the psychophysical measurement of corresponding points and our predictions. Following^43^, the evaluation was repeated considering two different ranges of eccentricity, *i.e.* [−1.6°, 1.6] and [−6°, 6°], in order to better characterize the difference between the central and the peripheral field of view. Regarding the receptive filed size, we computed the *rmse* between our prediction and the data reported in ref. 63. For a more detailed explanation, see Methods.

## References

[b1] Ohzawa I., Freeman R. D. & DeAngelis G. C. Stereoscopic depth discrimination in the visual cortex: Neurons ideally suited as disparity detectors. Science 249, 1037–1041 (1990). 2396096 10.1126/science.2396096

[b2] Qian N. Computing stereo disparity and motion with known binocular cell properties. Neural Computation 6 **(3)**, 390–404 (1994).

[b3] Gilaie-Dotan S., Ullman S., Kushnir T. & Malach R. Shape-selective stereo processing in human object-related visual areas. Human brain mapping 15, 67–79 (2002). 11835599 10.1002/hbm.10008 PMC6872042

[b4] Cottereau B. R., McKee S. P., Ales J. M. & Norcia A. M. Disparity-tuned population responses from human visual cortex. The Journal of Neuroscience 31, 954–965 (2011). 21248120 10.1523/JNEUROSCI.3795-10.2011 PMC3298090

[b5] Yang Z. & Purves D. A statistical explanation of visual space. Nature neuroscience 6, 632–640 (2003). 12754512 10.1038/nn1059

[b6] Adams W. J., Elder J. H., Graf E. W., Leyland J., Lugtigheid A. J. & Muryy A. The Southampton-York Natural Scenes (SYNS) dataset: Statistics of surface attitude. Scientific Reports 6 (2016). 10.1038/srep35805 PMC5080654 27782103

[b7] Field D. J. Relations between the statistics of natural images and the response properties of cortical cells. JOSA A 4, 2379–2394 (1987). 10.1364/josaa.4.002379 3430225

[b8] Simoncelli E. P. & Olshausen B. A. Natural image statistics and neural representation. Annual review of neuroscience 24, 1193–1216 (2001). 10.1146/annurev.neuro.24.1.1193 11520932

[b9] Geisler W. S. Visual perception and the statistical properties of natural scenes. Annu. Rev. Psychol. 59, 167–192 (2008). 17705683 10.1146/annurev.psych.58.110405.085632

[b10] Schreiber K. M., Hillis J. M., Filippini H. R., Schor C. M. & Banks M. S. The surface of the empirical horopter. Journal of Vision 8, 7 (2008). 10.1167/8.3.7 18484813

[b11] Tyler C. W. The horopter and binocular fusion. In CRC Press Inc., B. (ed.) Binocular vision(Nature Publishing Group, 1991).

[b12] Nakayama K. Human depth perception. Society of Photo-Optical Instrumentation Engineering Journal 120, 2–9 (1977).

[b13] von Helmholtz H. & Southall J. P. C. Helmholtz’s Treatise on Physiological Optics: Translated from the Third German Edition(Optical Society of America, 1925).

[b14] Sprague W. W., Cooper E. A., Tosić I. & Banks M. S. Stereopsis is adaptive for the natural environment. Science Advances 1, e1400254 (2015). 26207262 10.1126/sciadv.1400254 PMC4507831

[b15] Read J. C. A. & Cumming B. G. Understanding the cortical specialization for horizontal disparity. Neural Computation 16, 1983–2020 (2004). 15333204 10.1162/0899766041732440 PMC1382191

[b16] Liu Y., Bovik A. C. & Cormack L. K. Disparity statistics in natural scenes. Journal of Vision 8, 19 (2008). 10.1167/8.11.19 18831613

[b17] Cooper E. A. & Norcia A. M. Perceived depth in natural images reflects encoding of low-level luminance statistics. The Journal of Neuroscience 34, 11761–11768 (2014). 25164671 10.1523/JNEUROSCI.1336-14.2014 PMC4145177

[b18] Hibbard P. B. A statistical model of binocular disparity. Visual Cognition 15, 149–165 (2007).

[b19] Hibbard P. B. & Bouzit S. Stereoscopic correspondence for ambiguous targets is affected by elevation and fixation distance. Spatial vision 18, 399–411 (2005). 16167773 10.1163/1568568054389589

[b20] Burge J. & Geisler W. S. Optimal disparity estimation in natural stereo images. Journal of vision 14, 1–1, (2014). 10.1167/14.2.1 PMC3912897 24492596

[b21] Wexler M. & Ouarti N. Depth affects where we look. Current Biology 18, 1872–1876 (2008). 19062283 10.1016/j.cub.2008.10.059

[b22] Jansen L., Onat S. & König P. Influence of disparity on fixation and saccades in free viewing of natural scenes. Journal of Vision 9, 29 (2009). 10.1167/9.1.29 19271899

[b23] Wismeijer D., Erkelens C., van Ee R. & Wexler M. Depth cue combination in spontaneous eye movements. Journal of vision 10, 25 (2010). 10.1167/10.6.25 20884574

[b24] Land M. F. & Hayhoe M. In what ways do eye movements contribute to everyday activities? Vision research 41, 3559–3565 (2001). 11718795 10.1016/s0042-6989(01)00102-x

[b25] Howard I. P. & Rogers B. J. Seeing in Depth(I Porteous, Toronto, 2002).

[b26] Bohil C. J., Alicea B. & Biocca F. A. Virtual reality in neuroscience research and therapy. Nature reviews neuroscience 12, 752–762 (2011). 22048061 10.1038/nrn3122

[b27] Tweed D. & Vilis T. Geometric relations of eye position and velocity vectors during saccades. Vision Res. 30 **(1)**, 111–127 (1990). 2321357 10.1016/0042-6989(90)90131-4

[b28] Mok D., Ro A., Cadera W., Crawford J. D. & Vilis T. Rotation of listing’s plane during vergence. Vision Research 32, 2055–2064 (1992). 1304083 10.1016/0042-6989(92)90067-s

[b29] Tweed D. Visual-motor optimization in binocular control. Vision Res. 37 **(14)**, 1939–1951 (1997). 9274779 10.1016/s0042-6989(97)00002-3

[b30] Canessa A., Gibaldi A., Chessa M., Fato M., Solari F. & Sabatini S. P. A dataset of stereoscopic images and ground-truth disparity mimicking human fixations in peripersonal space. *Scientific Data*, doi: 10.1038/sdata.2017.34. 10.1038/sdata.2017.34 PMC5369322 28350382

[b31] Canessa A., Gibaldi A., Chessa M., Fato M., Solari F. & Sabatini S. P. Dryad Digital Repository. http://dx.doi.org/10.5061/dryad.6t8vq (2016).

[b32] Read J. C. A., Phillipson G. P. & Glennerster A. Latitude and longitude vertical disparities. Journal of Vision 9, 11–11 (2009). 10.1167/9.13.11 PMC2837276 20055544

[b33] Livingstone M. S. & Tsao D. Y. Receptive fields of disparity-selective neurons in macaque striate cortex. Nature Neuroscience 2, 825–832 (1999). 10461222 10.1038/12199

[b34] Prince S. J. D., Cumming B. G. & Parker A. J. Range and mechanism of encoding of horizontal disparity in macaque V1. J. Neurophysiol. 87, 209–221 (2002). 11784743 10.1152/jn.00466.2000

[b35] Durand J. B., Celebrini S. & Trotter Y. Neural bases of stereopsis across visual field of the alert macaque monkey. Cerebral Cortex 17, 1260–1273 (2007). 16908495 10.1093/cercor/bhl050

[b36] Samonds J. M., Potetz B. R. & Lee T. S. Relative luminance and binocular disparity preferences are correlated in macaque primary visual cortex, matching natural scene statistics. Proceedings of the National Academy of Sciences 109, 6313–6318 (2012). 10.1073/pnas.1200125109 PMC3341027 22474369

[b37] Read J. C. A bayesian approach to the stereo correspondence problem. Neural Computation 14, 1371–1392 (2002). 12020451 10.1162/089976602753712981

[b38] Hansard M. & Horaud R. Patterns of binocular disparity for a fixating observer. BVAI 308–317 (2007).

[b39] Brouwer A., Franz V. H. & Gegenfurtner K. R. Differences in fixations between grasping and viewing objects. Journal of Vision 9, 18 (2009). 10.1167/9.1.18 19271888

[b40] Desanghere L. & Marotta J. Graspability of objects affects gaze patterns during perception and action tasks. Experimental brain research 212, 177–187 (2011). 21597930 10.1007/s00221-011-2716-x

[b41] Juravle G., Velasco C., Salgado-Montejo A. & Spence C. The hand grasps the center, while the eyes saccade to the top of novel objects. Frontiers in Psychology 6, 633 (2015). 26052291 10.3389/fpsyg.2015.00633 PMC4441126

[b42] Schreiber K. M., Tweed D. B. & Schor C. M. The extended horopter: Quantifying retinal correspondence across changes of 3D eye position. Journal of Vision 6, 6 (2006). 10.1167/6.1.6 16489859

[b43] Hillis J. M. & Banks M. S. Are corresponding points fixed? Vision research 41, 2457–2473 (2001). 11483177 10.1016/s0042-6989(01)00137-7

[b44] Ogle K. N. Researches in binocular vision(Saunders, Philadelphia, 1950).

[b45] Fogt N. & Jones R. The effect of forced vergence on retinal correspondence. Vision research 38, 2711–2719 (1998). 9775320 10.1016/s0042-6989(97)00448-3

[b46] Grove P. M., Kaneko H. & Ono H. The backward inclination of a surface defined by empirical corresponding points. Perception 30, 411–429 (2001). 11383190 10.1068/p3091

[b47] Siderov J., Harwerth R. S. & Bedell H. E. Stereopsis, cyclovergence and the backwards tilt of the vertical horopter. Vision research 39, 1347–1357 (1999). 10343847 10.1016/s0042-6989(98)00252-1

[b48] Harrold A. L. & Grove P. M. Binocular correspondence and the range of fusible horizontal disparities in the central visual field. Journal of vision 15, 12–12 (2015). 10.1167/15.8.12 26114675

[b49] Cooper E. A., Burge J. & Banks M. S. The vertical horopter is not adaptable, but it may be adaptive. Journal of Vision 11, 20 (2011). 10.1167/11.3.20 PMC3804431 21447644

[b50] Ogle K. N. Disparity limits of stereopsis. AMA archives of ophthalmology 48, 50–60 (1952). 14932562 10.1001/archopht.1952.00920010053008

[b51] Blakemore C. The range and scope of binocular depth discrimination in man. The Journal of Physiology 211, 599–622 (1970). 5501054 10.1113/jphysiol.1970.sp009296 PMC1396079

[b52] Wilcox L. M. & Allison R. S. Coarse-fine dichotomies in human stereopsis. Vision research 49, 2653–2665 (2009). 19520102 10.1016/j.visres.2009.06.004

[b53] Krol J. D. & van der Grind W. A. Rehabilitation of a classical notion of panum’s fusional area. Perception 11, 615–619 (1982). 7186114 10.1068/p110615

[b54] Hampton D. R. & Kertesz A. E. The extent of panum’s area and the human cortical magnification factor. Perception 12, 161–165 (1983). 6657422 10.1068/p120161

[b55] Yeshurun Y. & Schwartz E. L. Cortical hypercolumn size determines stereo fusion limits. Biological cybernetics 80, 117–129 (1999). 10074690 10.1007/s004220050510

[b56] Schwartz E. L. Spatial mapping in the primate sensory projection: analytic structure and relevance to perception. Biological cybernetics 25, 181–194 (1977). 843541 10.1007/BF01885636

[b57] Koenderink J. J. & van Doorn A. J. Representation of local geometry in the visual system. Biological cybernetics 55, 367–375 (1987). 3567240 10.1007/BF00318371

[b58] Daniel P. & Whitteridge D. The representation of the visual field on the calcarine cortex in baboons and monkeys. In Journal of Physiology-LONDON vol. 148, 33–34 (,1959).

[b59] Rovamo J. & Virsu V. An estimation and application of the human cortical magnification factor. Experimental Brain Research 37, 495–510 (1979). 520439 10.1007/BF00236819

[b60] Smallman H. S. & MacLeod D. I. A. Size-disparity correlation in stereopsis at contrast threshold. JOSA A 11, 2169–2183 (1994). 7931758 10.1364/josaa.11.002169

[b61] Anzai A., Ohzawa I. & Freeman R. D. Neural mechanisms underlying binocular fusion and stereopsis: position vs. phase. Proceedings of the National Academy of Sciences 94, 5438–5443 (1997). 10.1073/pnas.94.10.5438 PMC24697 9144256

[b62] DeValois R. L. & DeValois K. K. Spatial vision. 14 (Oxford University Press, 1990).

[b63] Harvey B. M. & Dumoulin S. O. The relationship between cortical magnification factor and population receptive field size in human visual cortex: constancies in cortical architecture. The Journal of Neuroscience 31, 13604–13612 (2011). 21940451 10.1523/JNEUROSCI.2572-11.2011 PMC6623292

[b64] Cornell E. D., Macdougall H. G., Predebon J., Curthoys I. S. . Errors of binocular fixation are common in normal subjects during natural conditions. Optometry & Vision Science 80, 764–771 (2003). 14627944 10.1097/00006324-200311000-00014

[b65] Schor C. Fixation of disparity: a steady state error of disparity-induced vergence. American journal of optometry and physiological optics 57, 618–631 (1980). 7425085

[b66] London R. & Crelier R. S. Fixation disparity analysis: Sensory and motor approaches. Optometry-Journal of the American Optometric Association 77, 590–608 (2006). 10.1016/j.optm.2006.09.006 17157241

[b67] Masson G. S., Busettini C. & Miles F. A. Vergence eye movements in response to binocular disparity without depth perception. Nature 389, 283–286 (1997). 9305842 10.1038/38496

[b68] Cumming B. G. & Parker A. J. Responses of primary visual cortical neurons to binocular disparity without depth perception. Nature 389, 280–283 (1997). 9305841 10.1038/38487

[b69] Cloherty S. L., Hughes N. J., Hietanen M. A., Bhagavatula P. S., Goodhill G. J. Ibbotson M. R. Sensory experience modifies feature map relationships in visual cortex. eLife 5, e13911 (2016). 27310531 10.7554/eLife.13911 PMC4911216

[b70] Braddick O. J., Wattam-Bell J. & Atkinson J. Orientation-specific cortical responses develop in early infancy. Nature(1986). 10.1038/320617a0 3702994

[b71] Held R., Birch E. E. & Gwiazda J. Stereoacuity of human infants. Proceedings of the National Academy of Sciences 77, 5572–5574 (1980). 10.1073/pnas.77.9.5572 PMC350104 6933571

[b72] Mitchell D. E. & Timney B. Postnatal development of function in the mammalian visual system. Comprehensive Physiology(1984).

[b73] Thorn F., Gwiazda J., Cruz A. A., Bauer J. A. & Held R. The development of eye alignment, convergence, and sensory binocularity in young infants. Invest. Ophthalmol. Vis. Sci. 35, 544–553 (1994). 8113005

[b74] Birch E. E., Gwiazda J. & Held R. Stereoacuity development for crossed and uncrossed disparities in human infants. Vis. Res. 22, 507–513 (1982). 6981241 10.1016/0042-6989(82)90108-0

[b75] Açk A., Sarwary A., Schultze-Kraft R., Onat S. & König P. Developmental changes in natural viewing behavior: bottom-up and top-down differences between children, young adults and older adults. Frontiers in psychology 1, 207 (2010). 21833263 10.3389/fpsyg.2010.00207 PMC3153813

[b76] Fox R. & Patterson R. Depth separation and lateral interference. Perception & Psychophysics 30, 513–520 (1981). 7335446 10.3758/bf03202004

[b77] Burge J., Fowlkes C. C. & Banks M. S. Natural-scene statistics predict how the figure–ground cue of convexity affects human depth perception. The Journal of Neuroscience 30, 7269–7280 (2010). 20505093 10.1523/JNEUROSCI.5551-09.2010 PMC3062505

[b78] Hinkle D. A. & Connor C. E. Quantitative characterization of disparity tuning in ventral pathway area V4. Journal of neurophysiology 94, 2726–2737 (2005). 15987762 10.1152/jn.00341.2005

[b79] Hoffman D. M., Girshick A. R., Akeley K. & Banks M. S. Vergence–accommodation conflicts hinder visual performance and cause visual fatigue. Journal of vision 8, 33–33 (2008). 10.1167/8.3.33 PMC2879326 18484839

[b80] Watt S. J., Akeley K., Ernst M. O. & Banks M. S. Focus cues affect perceived depth. Journal of vision 5, 7–7 (2005). 10.1167/5.10.7 PMC2667386 16441189

[b81] Shibata T., Kim J., Hoffman D. M. & Banks M. S. The zone of comfort: Predicting visual discomfort with stereo displays. Journal of vision 11, 11–11 (2011). 10.1167/11.8.11 PMC3369815 21778252

[b82] Bahil A. T., Adler D. & Stark L. Most naturally occurring human saccades have magnitudes of 15 degrees or less. Investigative Ophthalmology & Visual Science 14, 468–469 (1975). 1132942

[b83] Stahl J. S. Eye-head coordination and the variation of eye-movement accuracy with orbital eccentricity. Experimental brain research 136, 200–210 (2001). 11206282 10.1007/s002210000593

[b84] Solari F., CHessa M. & Sabatini S. P. Natural perception in dynamic stereoscopic augmented reality environments. Displays 34, 142–152 (2013).

[b85] Banks M. S., Read J. C. A., Allison R. S. & Watt S. J. Stereoscopy and the human visual system. SMPTE motion imaging journal 121, 24–43 (2012). 23144596 10.5594/j18173 PMC3490636

[b86] Chessa M., Solari F. & Sabatini S. P. Virtual reality to simulate visual tasks for robotic systems. In, J.-J. K. E. (ed.) Virtual Reality 71–92 (Citeseer, 2010).

[b87] Scharstein D. & Szeliski R. A taxonomy and evaluation of dense two-frame stereo correspondence algorithms. International journal of computer vision 47, 7–42 (2002).

[b88] Hunter D. W. & Hibbard P. B. Distribution of independent components of binocular natural images. Journal of vision 15, 6–6 (2015). 10.1167/15.13.6 26381837

[b89] Liu Y., Cormack L. K. & Bovik A. C. Dichotomy between luminance and disparity features at binocular fixations. Journal of vision 10, 23 (2010). 10.1167/10.12.23 21047755

[b90] Erkelens C. J. & van Ee R. d. A computational model of depth perception based on headcentric disparity. Vision Research 38, 2999–3018 (1998). 9797995 10.1016/s0042-6989(98)00084-4

[b91] Schreiber K., Crawford J. D., Fetter M. & Tweed D. The motor side of depth vision. Nature 410, 819–822 (2001). 11298450 10.1038/35071081

[b92] Gibaldi A., Canessa A., Chessa M. A., Solari F. & Sabatini S. P. A neural model for coordinated control of horizontal and vertical alignment of the eyes in three-dimensional space *In Biomedical robotics and biomechatronics (BioRob), 2012 4th IEEE RAS & EMBS international conference on* 955–960 (2012).

[b93] Gibaldi A., Vanegas M., Pex P. J. & Maiello G. Evaluation of the Tobii EyeX Eye tracking controller and Matlab toolkit for research. Behavior Research Methods 1–24 (2016). 27401169 10.3758/s13428-016-0762-9 PMC5429393

[b94] Svede A., Treija E., Jaschinski W. & Krumina G. Monocular versus binocular calibrations in evaluating fixation disparity with a video-based eye-tracker. Perception 44, 1110–1128 (2015). 26562925 10.1177/0301006615596886

[b95] Held R. T. & Banks M. S. Misperceptions in stereoscopic displays: a vision science perspective. *Proceedings of the 5th symposium on Applied perception in graphics and visualization* **23–32** (2008). 10.1145/1394281.1394285 PMC3966488 24683290

[b96] Cramér H. Mathematical methods of statistics vol. 9 (Princeton university press, 1945).

[b97] Ekström J. Mahalanobis’ distance beyond normal distributions. UCLA Statistics Preprints 624 (2011).

[b98] Jones J. P. & Palmer L. A. An evaluation of the two-dimensional Gabor filter model of simple receptive fields in cat striate cortex. Journal of neurophysiology 58, 1233–1258 (1987). 3437332 10.1152/jn.1987.58.6.1233

[b99] Gautier J. & Le Meur O. A time-dependent saliency model combining center and depth biases for 2D and 3D viewing conditions. Cognitive Computation 4, 141–156 (2012).

