# Supplementary material for: The Active Side of Stereopsis: Fixation Strategy and Adaptation to Natural Environments
Source: Sci Rep. 2017 Mar 20;7:44800. doi: 10.1038/srep44800 (PMC5357847; doi:10.1038/srep44800)
Supplement: Supplementary Material [file srep44800-s1.pdf]

The Active Side of Stereopsis: Fixation Strategy and Adaptation to Natural Environments

Agostino Gibaldi[1,*], Andrea Canessa[1], and Silvio P. Sabatini[1]

[1]Physical Structure of Perception and Computation Group, Department of Informatics, Bioengineering, Robotics and System Engineering, University of Genoa, 16145, Genoa, Italy
[*]agostino.gibaldi@unige.it

ABSTRACT

Depth perception in near viewing strongly relies on the interpretation of binocular retinal disparity to obtain stereopsis. Statistical regularities of retinal disparities have been claimed to greatly impact on the neural mechanisms that underlie binocular vision, both to facilitate perceptual decisions and to reduce computational load. In order to assess how fixation strategy can finely conditions the statistics of the disparity a novel and unconventional approach has been designed. The approach integrates accurate realistic three-dimensional models of natural scenes with binocular eye movement recording, allowing accurate ground-truth statistics of retinal disparity experienced by a subject in near viewing. Our results evidence how the organization of human binocular visual system is finely adapted to the disparity statistics that quantitatively distinguish actual fixations, revealing a novel role of the active fixation strategy over the binocular visual functionality. This suggests an ecological explanation of the intrinsic preference of the mechanism of stereopsis for a close central object surrounded by a far background, as an early binocular aspect of the figure-ground segregation process.

Supplementary Materials

Disparity Patterns and Empirical Horopter for the Single Subjects - Figure S1 shows the retinotopic median horizontal disparity (top), the patterns of disparity variability (middle), and the horizontal and vertical horopter (bottom), separately for the four subjects involved in the experiment.

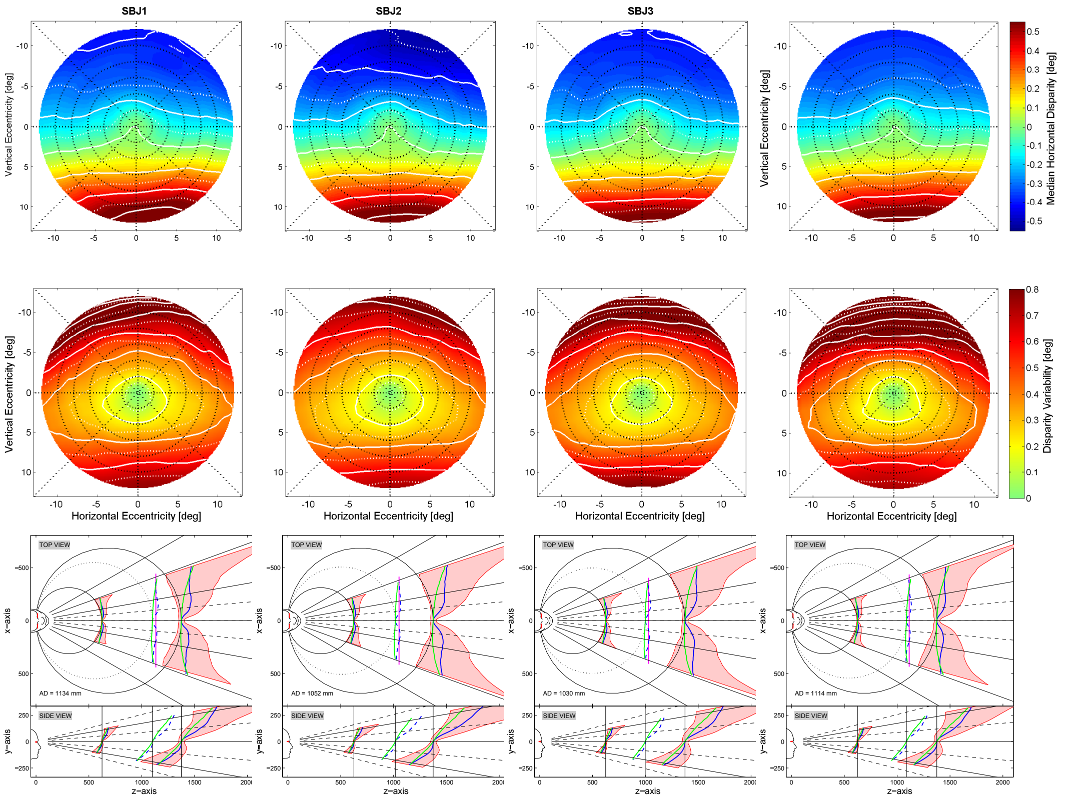

Figure S1. Individual subjects' data. Horizontal median disparity pattern and standard deviation (top rows), as in Fig. 3, and top and side views of the empirical horopter and Panum's fusional area (bottom row), as in Fig. 5, represented for each subject, separately.

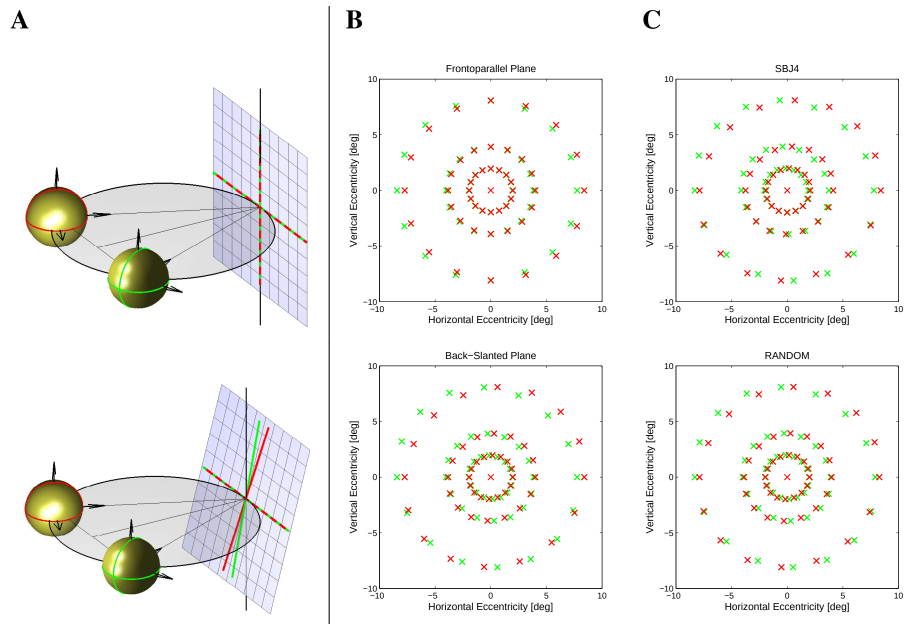

Figure S2. **A. Geometry of viewing posture while looking a plane.** Sketches of the fixating eye system, showing the eyeballs and the horizontal and vertical meridians for the left (red) and right (green) eye, projected on the fixed plane (gray mesh). The plane is at 400 mm of distance from the observer (vergence angle $\approx 8.5°$), with straight-ahead binocular gaze direction, and fixation point lying on its center, with a tilt of 0° (top) and 15° (bottom). The sketch represents also the geometric horopters, as the horizontal Vieth-Müller circle (black) and vertical horopter (black vertical line). **B. Empirical Retinal Corresponding Points Generated by a Frontoparallel Plane.** Pattern of empirical corresponding points represented in the first 10° of visual field eccentricity, which would be generated by a frontoparallel plane (top) or by a back-slanted plane (bottom). **C. Empirical Corresponding Points Generated on Observer's Fixations.** Pattern of empirical corresponding points represented in the first 10° of visual field eccentricity, which would be generated by the median disparity computed on subjects' (top) or random (bottom) fixations.

Retinal Empirical Correspondence across the Field of View. - The empirical correspondence has been commonly studied along the horizontal[14,36–38,40,42] and vertical[13,15,34,37,40–42] meridians of the field of view, as cardinal directions of human vision. The definition of the Hering-Hillebrand (horizontal) and Helmholtz shear (vertical) deviation, does not imply that retinal deviations occur along the two meridians only, but they encompass the whole field of view[15,39]. Thus, we extended the comparison to the whole (central) field of view, and compared our results to the disparity pattern projected by a frontoparallel and a back-slanted plane, as in[15] (see Fig. 2A). Both the patterns of empirical corresponding points computed by our disparity statistics for of subjects' and random fixations (see Fig. 2B, right) are more similar to a pattern produced by a back-slanted plane than that produced by a frontoparallel plane (see Fig. 2B, left). Again, the subjects' pattern presents a higher similarity with those measured in humans ($rmse = 0.14°$), than that obtained by random fixations ($rmse = 0.15°$).

The 3D shape of the empirical horopter. - The empirical corresponding point over the whole field of view (first 10° of eccentricity) have been exploited to compute the 3D shape of the empirical horopter (see Fig. S3, derived by the disparity statistics for subjects' fixations. The horopter has been computed as the surfaces that project with minimum retinal disparity to the pairs of empirical corresponding points[35]. It is evident how random fixations (B) result in a 3D shape which is smooth and continuous moving from the center to the periphery of the field of view, while the horopter derived by subjects' fixation is characterized by a close central bump surrounded by a further background.

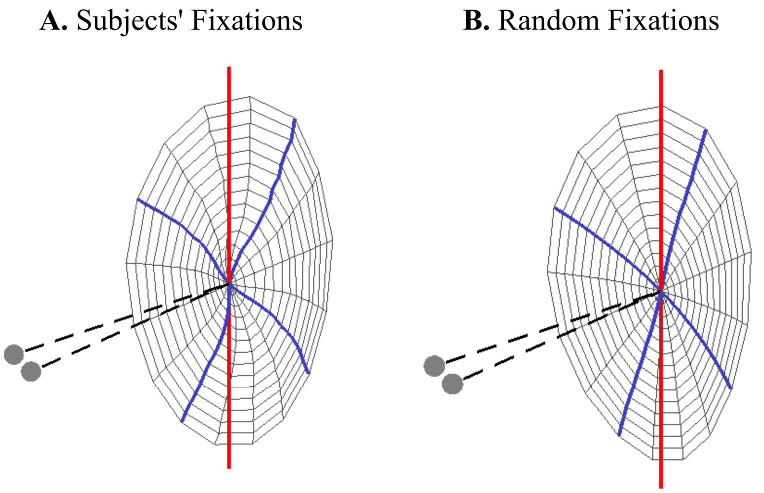

Figure S3. **A. 3D shape of the empirical horopter for subjects' fixations.** The 3D empirical horopter, derived by the disparity statistics for subjects' fixations, computed as the surfaces that project with minimum retinal disparity to the pairs of empirical corresponding points. The optical axes (black dashed lines) originating from the eyeballs, intersect at the fixation point. The observer is fixating straight-ahead, with a vergence angle of $7°$, *i.e.* at a distance of $\approx$ 500 mm. The empirical horopter is represented within the first $10°$ of eccentricity (gray mesh), highlighting the horizontal and vertical meridians (blue lines), and showing the geometric vertical horopter (red line). The observer is fixating straight-ahead, with a vergence angle of $7°$, *i.e.* at a distance of $\approx$ 500 mm. **B. 3D shape of the empirical horopter for random fixations.** The 3D empirical horopter, derived by the disparity statistics for subjects' fixations, computed as in Panel A.

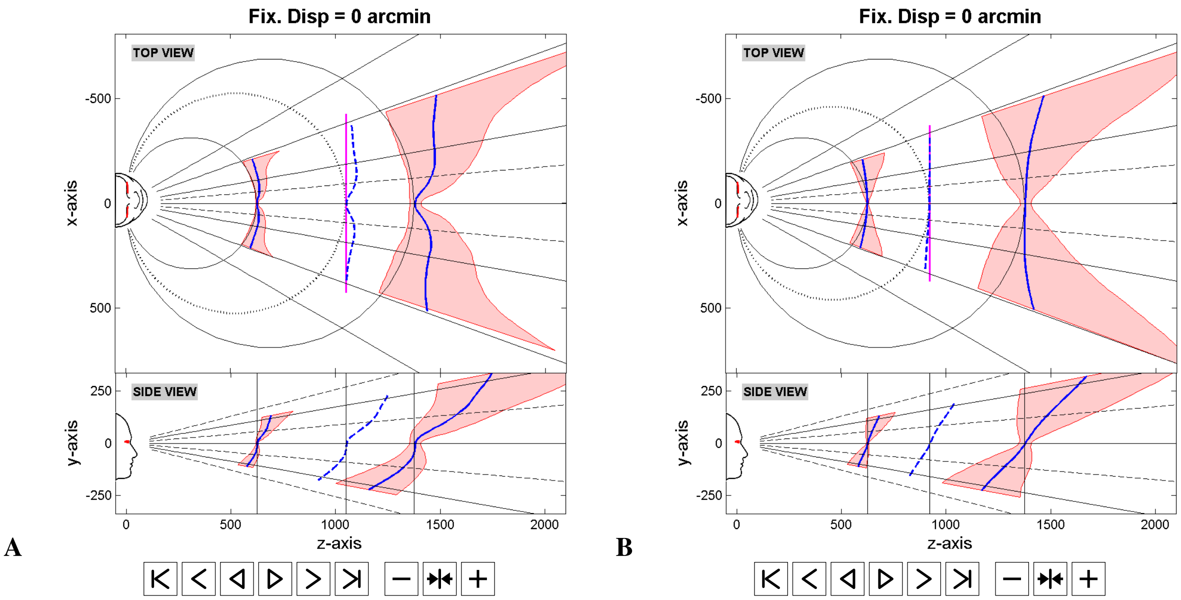

Figure S4. **A. Empirical horopters and Panum's areas with fixation disparity.** Representation of the horizontal (top) and vertical (bottom) horopter, with the associate Panum's area, for subjects' (A) and random (B) fixations, as in Fig. 5. The graphs display the variation of the empirical horopter and Panum's area when a fixation disparity varying from zero to 40 arcmin[58] is introduced.

Fixation disparity and Panum's fusional area - Fig. S4 shows the horizontal (top) and vertical (bottom) horopter, with the associate Panum's area (pink area), for subjects' (A) and random (B) fixations, as in Fig. 5, but simulating a fixation disparity. We assumed a positive fixation disparity, as commonly measured in normal subjects[58], and we simulated the fixation disparity varying from zero to 40 arcmin. The increasing fixation disparity has the effect of shifting the empirical horopter closer to the geometric horopter. This effect is more convenient in subjects' fixations, since it reduces the global disparity experienced by the subject, while a local adaptation of the retinal correspondence accounts for a null perceived disparity at fixation.

References

1. Ohzawa, I., Freeman, R. D. & DeAngelis, G. C. Stereoscopic depth discrimination in the visual cortex: Neurons ideally suited as disparity detectors. *Science* **249**, 1037–1041 (1990).
2. Qian., N. Computing stereo disparity and motion with known binocular cell properties. *Neural Computation* **6(3)**, 390–404 (1994).
3. Gilaie-Dotan, S., Ullman, S., Kushnir, T. & Malach, R. Shape-selective stereo processing in human object-related visual areas. *Human brain mapping* **15**, 67–79 (2002).
4. Cottereau, B. R., McKee, S. P., Ales, J. M. & Norcia, A. M. Disparity-tuned population responses from human visual cortex. *The Journal of Neuroscience* **31**, 954–965 (2011).
5. Field, D. J. Relations between the statistics of natural images and the response properties of cortical cells. *JOSA A* **4**, 2379–2394 (1987).
6. Simoncelli, E. P. & Olshausen, B. A. Natural image statistics and neural representation. *Annual review of neuroscience* **24**, 1193–1216 (2001).
7. Geisler, W. S. Visual perception and the statistical properties of natural scenes. *Annu. Rev. Psychol.* **59**, 167–192 (2008).
8. Read, J. C. A. & Cumming, B. G. Understanding the cortical specialization for horizontal disparity. *Neural Computation* **16**, 1983–2020 (2004).
9. Liu, Y., Bovik, A. C. & Cormack, L. K. Disparity statistics in natural scenes. *Journal of Vision* **8**, 19 (2008).
10. Cooper, E. A. & Norcia, A. M. Perceived depth in natural images reflects encoding of low-level luminance statistics. *The Journal of Neuroscience* **34**, 11761–11768 (2014).
11. Yang, Z. & Purves, D. A statistical explanation of visual space. *Nature neuroscience* **6**, 632–640 (2003).
12. Adams, W. J., Elder, J. H., Graf, E. W., Leyland, J., Lugtigheid, A. J. & Muryy, A. The Southampton-York Natural Scenes (SYNS) dataset: Statistics of surface attitude. *Scientific Reports* **6** (2016).
13. Sprague, W. W., Cooper, E. A., Tosić, I. & Banks, M. S. Stereopsis is adaptive for the natural environment. *Science Advances* **1**, e1400254 (2015).
14. Hillis, J. M. & Banks, M. S. Are corresponding points fixed? *Vision research* **41**, 2457–2473 (2001).
15. Schreiber, K. M., Hillis, J. M., Filippini, H. R., Schor, C. M. & Banks, M. S. The surface of the empirical horopter. *Journal of Vision* **8**, 7 (2008).
16. Land, M. F. & Hayhoe, M. In what ways do eye movements contribute to everyday activities? *Vision research* **41**, 3559–3565 (2001).
17. Hibbard, P. B. A statistical model of binocular disparity. *Visual Cognition* **15**, 149–165 (2007).
18. Howard, I. P. & Rogers, B. J. *Seeing in Depth* (I Porteous, Toronto, 2002).
19. Tweed, D. & Vilis, T. Geometric relations of eye position and velocity vectors during saccades. *Vision Res.* **30(1)**, 111–127 (1990).
20. Mok, D., Ro, A., Cadera, W., Crawford, J. D. & Vilis, T. Rotation of listing's plane during vergence. *Vision Research* **32**, 2055–2064 (1992).
21. Tweed, D. Visual-motor optimization in binocular control. *Vision Res.* **37(14)**, 1939–1951 (1997).
22. Livingstone, M. S. & Tsao, D. Y. Receptive fields of disparity-selective neurons in macaque striate cortex. *Nature Neuroscience* **2**, 825–832 (1999).
23. Prince, S. J. D., Cumming, B. G. & Parker, A. J. Range and mechanism of encoding of horizontal disparity in macaque V1. *J. Neurophysiol.* **87**, 209–221 (2002).
24. Durand, J. B., Celebrini, S. & Trotter, Y. Neural bases of stereopsis across visual field of the alert macaque monkey. *Cerebral Cortex* **17**, 1260–1273 (2007).
25. Samonds, J. M., Potetz, B. R. & Lee, T. S. Relative luminance and binocular disparity preferences are correlated in macaque primary visual cortex, matching natural scene statistics. *Proceedings of the National Academy of Sciences* **109**, 6313–6318 (2012).
26. Read, J. C. A bayesian approach to the stereo correspondence problem. *Neural Computation* **14**, 1371–1392 (2002).
27. Hansard, M. & Horaud, R. Patterns of binocular disparity for a fixating observer. *BVAI* 308–317 (2007).

28. Wexler, M. & Ouarti, N. Depth affects where we look. *Current Biology* **18**, 1872–1876 (2008).

29. Jansen, L., Onat, S. & König, P. Influence of disparity on fixation and saccades in free viewing of natural scenes. *Journal of Vision* **9**, 29 (2009).

30. Wismeijer, D., Erkelens, C., van Ee, R. & Wexler, M. Depth cue combination in spontaneous eye movements. *Journal of vision* **10**, 25 (2010).

31. Brouwer, A., Franz, V. H. & Gegenfurtner, K. R. Differences in fixations between grasping and viewing objects. *Journal of Vision* **9**, 18 (2009).

32. Desanghere, L. & Marotta, J. "graspability" of objects affects gaze patterns during perception and action tasks. *Experimental brain research* **212**, 177–187 (2011).

33. Juravle, G., Velasco, C., Salgado-Montejo, A. & Spence, C. The hand grasps the center, while the eyes saccade to the top of novel objects. *Frontiers in Psychology* **6**, 633 (2015).

34. Tyler, C. W. The horopter and binocular fusion. In CRC Press Inc., B. (ed.) *Binocular vision* (Nature Publishing Group, 1991).

35. Schreiber, K. M., Tweed, D. B. & Schor, C. M. The extended horopter: Quantifying retinal correspondence across changes of 3d eye position. *Journal of Vision* **6**, 6 (2006).

36. Ogle, K. N. *Researches in binocular vision* (Saunders, Philadelphia, 1950).

37. Nakayama, K. Human depth perception. *Society of Photo-Optical Instrumentation Engineering Journal* **120**, 2–9 (1977).

38. Fogt, N. & Jones, R. The effect of forced vergence on retinal correspondence. *Vision research* **38**, 2711–2719 (1998).

39. Grove, P. M., Kaneko, H. & Ono, H. The backward inclination of a surface defined by empirical corresponding points. *Perception* **30**, 411–429 (2001).

40. von Helmholtz, H. & Southall, J. P. C. *Helmholtz's Treatise on Physiological Optics: Translated from the Third German Edition* (Optical Society of America, 1925).

41. Siderov, J., Harwerth, R. S. & Bedell, H. E. Stereopsis, cyclovergence and the backwards tilt of the vertical horopter. *Vision research* **39**, 1347–1357 (1999).

42. Harrold, A. L. & Grove, P. M. Binocular correspondence and the range of fusible horizontal disparities in the central visual field. *Journal of vision* **15**, 12–12 (2015).

43. Cooper, E. A., Burge, J. & Banks, M. S. The vertical horopter is not adaptable, but it may be adaptive. *Journal of Vision* **11**, 20 (2011).

44. Ogle, K. N. Disparity limits of stereopsis. *AMA archives of ophthalmology* **48**, 50–60 (1952).

45. Blakemore, C. The range and scope of binocular depth discrimination in man. *The Journal of Physiology* **211**, 599–622 (1970).

46. Wilcox, L. M. & Allison, R. S. Coarse-fine dichotomies in human stereopsis. *Vision research* **49**, 2653–2665 (2009).

47. Krol, J. D. & van der Grind, W. A. Rehabilitation of a classical notion of panum's fusional area. *Perception* **11**, 615–619 (1982).

48. Hampton, D. R. & Kertesz, A. E. The extent of panum's area and the human cortical magnification factor. *Perception* **12**, 161–165 (1983).

49. Yeshurun, Y. & Schwartz, E. L. Cortical hypercolumn size determines stereo fusion limits. *Biological cybernetics* **80**, 117–129 (1999).

50. Schwartz, E. L. Spatial mapping in the primate sensory projection: analytic structure and relevance to perception. *Biological cybernetics* **25**, 181–194 (1977).

51. Koenderink, J. J. & van Doorn, A. J. Representation of local geometry in the visual system. *Biological cybernetics* **55**, 367–375 (1987).

52. Daniel, P. & Whitteridge, D. The representation of the visual field on the calcarine cortex in baboons and monkeys. In *Journal of Physiology-LONDON*, vol. 148, 33–34 (Cambridge Univ. Press 40 West 20th Street, NEW YORK, 1959).

53. Rovamo, J. & Virsu, V. An estimation and application of the human cortical magnification factor. *Experimental Brain Research* **37**, 495–510 (1979).

54. Smallman, H. S. & MacLeod, D. I. A. Size-disparity correlation in stereopsis at contrast threshold. *JOSA A* **11**, 2169–2183 (1994).

55. Anzai, A., Ohzawa, I. & Freeman, R. D. Neural mechanisms underlying binocular fusion and stereopsis: position vs. phase. *Proceedings of the National Academy of Sciences* **94**, 5438–5443 (1997).

56. DeValois, R. L. & DeValois, K. K. *Spatial vision*. 14 (Oxford University Press, 1990).

57. Harvey, B. M. & Dumoulin, S. O. The relationship between cortical magnification factor and population receptive field size in human visual cortex: constancies in cortical architecture. *The Journal of Neuroscience* **31**, 13604–13612 (2011).

58. Cornell, E. D., Macdougall, H. G., Predebon, J., Curthoys, I. S. *et al.* Errors of binocular fixation are common in normal subjects during natural conditions. *Optometry & Vision Science* **80**, 764–771 (2003).

59. Gibaldi, A., Vanegas, M., Pex, P. J. & Maiello, G. Evaluation of the Tobii EyeX Eye tracking controller and Matlab toolkit for research. *Behavior Research Methods*, 1–24 (2016).

60. Schor, C. Fixation of disparity: a steady state error of disparity-induced vergence. *American journal of optometry and physiological optics* **57**, 618–631 (1980).

61. London, R. & Crelier, R. S. Fixation disparity analysis: Sensory and motor approaches. *Optometry-Journal of the American Optometric Association* **77**, 590–608 (2006).

62. Masson, G. S., Busettini, C. & Miles, F. A. Vergence eye movements in response to binocular disparity without depth perception. *Nature* **389**, 283–286 (1997).

63. Cumming, B. G. & Parker, A. J. Responses of primary visual cortical neurons to binocular disparity without depth perception. *Nature* **389**, 280–283 (1997).

64. Cloherty, S. L. *et al.* Sensory experience modifies feature map relationships in visual cortex. *eLife* **5**, e13911 (2016).

65. Braddick, O. J., Wattam-Bell, J. & Atkinson, J. Orientation-specific cortical responses develop in early infancy. *Nature* (1986).

66. Held, R., Birch, E. E. & Gwiazda, J. Stereoacuity of human infants. *Proceedings of the National Academy of Sciences* **77**, 5572–5574 (1980).

67. Mitchell, D. E. & Timney, B. Postnatal development of function in the mammalian visual system. *Comprehensive Physiology* (1984).

68. Thorn, F., Gwiazda, J., Cruz, A. A., Bauer, J. A. & Held, R. The development of eye alignment, convergence, and sensory binocularity in young infants. *Invest. Ophthalmol. Vis. Sci.* **35**, 544–553 (1994).

69. Birch, E. E., Gwiazda, J. & Held, R. Stereoacuity development for crossed and uncrossed disparities in human infants. *Vis. Res.* **22**, 507–513 (1982).

70. Açık, A., Sarwary, A., Schultze-Kraft, R., Onat, S. & König, P. Developmental changes in natural viewing behavior: bottom-up and top-down differences between children, young adults and older adults. *Frontiers in psychology* **1**, 207 (2010).

71. Hinkle, D. A. & Connor, C. E. Quantitative characterization of disparity tuning in ventral pathway area V4. *Journal of neurophysiology* **94**, 2726–2737 (2005).

72. Burge, J., Fowlkes, C. C. & Banks, M. S. Natural-scene statistics predict how the figure–ground cue of convexity affects human depth perception. *The Journal of Neuroscience* **30**, 7269–7280 (2010).

73. Gautier, J. & Le Meur, O. A time-dependent saliency model combining center and depth biases for 2d and 3d viewing conditions. *Cognitive Computation* **4**, 141–156 (2012).

74. Scharstein, D. & Szeliski, R. A taxonomy and evaluation of dense two-frame stereo correspondence algorithms. *International journal of computer vision* **47**, 7–42 (2002).

75. Hunter, D. W. & Hibbard, P. B. Distribution of independent components of binocular natural images. *Journal of vision* **15**, 6–6 (2015).

76. Liu, Y., Cormack, L. K. & Bovik, A. C. Dichotomy between luminance and disparity features at binocular fixations. *Journal of vision* **10**, 23 (2010).

77. Chessa, M., Solari, F. & Sabatini, S. P. Virtual reality to simulate visual tasks for robotic systems. In), J.-J. K. E. (ed.) *Virtual Reality*, 71–92 (Citeseer, 2010).

78. Solari, F., CHessa, M. & Sabatini, S. P. Natural perception in dynamic stereoscopic augmented reality environments. *Displays* **34**, 142–152 (2013).

79. Erkelens, C. J. & van Ee, R. d. A computational model of depth perception based on headcentric disparity. *Vision Research* **38**, 2999–3018 (1998).

80. Schreiber, K., Crawford, J. D., Fetter, M. & Tweed, D. The motor side of depth vision. *Nature* **410**, 819–822 (2001).

81. Svede, A., Treija, E., Jaschinski, W. & Krumina, G. Monocular versus binocular calibrations in evaluating fixation disparity with a video-based eye-tracker. *Perception* **44**, 1110–1128 (2015).

82. Cramér, H. *Mathematical methods of statistics*, vol. 9 (Princeton university press, 1945).

83. Ekström, J. Mahalanobis' distance beyond normal distributions. *UCLA Statistics Preprints* **624** (2011).

84. Jones, J. P. & Palmer, L. A. An evaluation of the two-dimensional gabor filter model of simple receptive fields in cat striate cortex. *Journal of neurophysiology* **58**, 1233–1258 (1987).

85. Held, R. T. & Banks, M. S. Misperceptions in stereoscopic displays: a vision science perspective. *Proceedings of the 5th symposium on Applied perception in graphics and visualization* **23–32**, (2008).

86. Fox, R. & Patterson, R. Depth separation and lateral interference. *Perception & Psychophysics* **30**, 513–520, (1981).

87. Banks, M. S., Read, J. C. A., Allison, R. S. & Watt, S. J. Stereoscopy and the human visual system. *SMPTE motion imaging journal* **121**, 24–43 (2012).

88. Bahil, A. T., Adler, D. & Stark, L. Most naturally occurring human saccades have magnitudes of 15 degrees or less. *Investigative Ophthalmology & Visual Science* **14**, 468–469 (1975).

89. Stahl, J. S. Eye-head coordination and the variation of eye-movement accuracy with orbital eccentricity. *Experimental brain research* **136**, 200–210 (2001).

90. Watt, S. J., Akeley, K., Ernst, M. O. & Banks, M. S. Focus cues affect perceived depth. *Journal of vision* **5**, 7–7 (2005).

91. Hoffman, D. M., Girshick, A. R., Akeley, K. & Banks, M. S. Vergence–accommodation conflicts hinder visual performance and cause visual fatigue. *Journal of vision* **8**, 33–33 (2008).

92. Shibata, T., Kim, J., Hoffman, D. M. & Banks, M. S. The zone of comfort: Predicting visual discomfort with stereo displays. *Journal of vision* **11**, 11–11 (2011).

93. Hibbard, P. B. & Bouzit, S. Stereoscopic correspondence for ambiguous targets is affected by elevation and fixation distance. *Spatial vision* **18**, 399–411, (2005).

94. Burge, J. & Geisler, W. S. Optimal disparity estimation in natural stereo images. *Journal of vision* **14**, 1–1, (2014).

95. Bohil, C. J., Alicea, B. & Biocca, F. A. Virtual reality in neuroscience research and therapy. *Nature reviews neuroscience* **12**, 752–762 (2011).

96. Read, J. C. A., Phillipson, G. P. & Glennerster, A. Latitude and longitude vertical disparities. *Journal of Vision* **9**, 11–11 (2009).
